# Pharmacological treatments and clinical events in newly diagnosed heart failure patients stratified by ejection fraction in Japan

**DOI:** 10.1038/s41598-026-58004-0

**Published:** 2026-06-13

**Authors:** Naoki Sato, Suguru Okami, Kanae Yoshikawa-Ryan, Satoshi Yamashita, Ryohei Kobayashi, Yoshifumi Arita, Seok-Won Kim, Rachel Knapp, Katsiaryna Holl, Gregg C. Fonarow

**Affiliations:** 1 Kawaguchi Cardiovascular and Respiratory Hospital, Kawaguchi, Japan; 2 Medical Affairs & Pharmacovigilance, Bayer Yakuhin Ltd., 2-4-9 Umeda, Kita-ku, Osaka, 530-0001 Japan; 3 IQVIA Solutions Japan G.K., Tokyo, Japan; 4 Global Medical and Evidence, Bayer AG, Berlin, Germany; 5 Division of Cardiology, UCLA Medical Center, Los Angeles, USA

**Keywords:** Heart failure, Drug therapy, Mortality, Outcomes, Left-ventricular ejection fraction, Epidemiology, Cardiology, Diseases, Medical research

## Abstract

There is a limited understanding of the uptake of pharmacological treatments and incidence of clinical events in newly diagnosed heart failure (HF) patients, stratified by left-ventricular ejection fraction (LVEF) in contemporary clinical settings in Japan. A retrospective cohort study was conducted using a nationwide Japanese hospital database to evaluate the patterns of HF medications and clinical events in adult patients with a first confirmed HF diagnosis from January 1, 2020–July 31, 2023 (n = 16,001). Fine-Gray sub-distribution hazard models were applied to assess factors associated with HF medication initiations and clinical events. Overall, 5473 (34.2%), 3053 (19.1%), and 7475 (46.7%) patients with HF with reduced ejection fraction, mildly reduced ejection fraction (HFmrEF), and preserved ejection fraction (HFpEF) were included, respectively. Within the first 6 months, prescription rates of HF medications were: angiotensin-converting enzyme inhibitors, angiotensin-receptor blockers, or angiotensin receptor blocker-neprilysin inhibitor (65.6%), beta-blockers (57.6%), mineralocorticoid antagonists (55.7%), and sodium-glucose cotransporter-2 inhibitors (33.2%), with the lowest rates in HFpEF patients. The increase in prescription rates between 6 and 12 months was modest across all medication classes. One-year incidence rates per 100 person-years (95% confidence interval) of in-hospital all-cause mortality and in-hospital cardiovascular death were 23.0 (22.1–24.0) and 16.6 (15.8–17.4), respectively. HFmrEF and HFpEF were associated with lower hazards of treatment initiation for most HF medications, whereas the risks of in-hospital all-cause mortality and in-hospital cardiovascular death did not differ significantly across all LVEF subtypes. The findings underscore the suboptimal uptake of pharmacological treatments, despite the poor prognosis of newly diagnosed HF patients. The disparities in initiations of recommended treatments reaffirm the importance of properly implementing evidence-based therapies within each LVEF subtype.

**Supplementary Information:**

The online version contains supplementary material available at 10.1038/s41598-026-58004-0.

## Introduction

Heart failure (HF) is a clinical syndrome caused by structural and/or functional abnormalities of the heart, resulting in impaired ventricular filling or ejection of blood, and associated symptoms such as dyspnea, edema, fatigue, and reduced exercise tolerance^1^. HF continues to pose a significant global health burden, affecting more than 64.3 million individuals worldwide^2^. Along with an aging population and an increasing burden of hypertension, obesity, and diabetes, the number of aging patients with HF is expected to grow in the coming years^3,4^.

HF is classified into three categories according to the left ventricular ejection fraction (LVEF): HF with reduced ejection fraction (HFrEF), HF with mildly reduced ejection fraction (HFmrEF), and HF with preserved ejection fraction (HFpEF)^1^. Whilst various pharmacological treatment options have been approved with a Class I guideline recommendation for use in patients with HFrEF, fewer options have been systematically recommended for HFmrEF or HFpEF^5,6^. In Japan, a four-pillar approach utilizing angiotensin-converting enzyme inhibitors (ACEis)/angiotensin-receptor blockers (ARBs) or angiotensin receptor blocker-neprilysin inhibitor (ARNI), mineralocorticoid receptor antagonists (MRAs), beta blockers (BBs), and sodium glucose cotransporter-2 inhibitors (SGLT2is) is recommended for HFrEF patients^7^. For HFmrEF and HFpEF, recommended treatments are limited to the administration of diuretics for congestion and SGLT2is.

Continuous monitoring of the progression of HF as a trajectory is important to both enable the early detection of HF and facilitate appropriate therapeutic interventions and care. The guidelines emphasize the importance of evaluating symptomatic HF by considering dynamic changes such as new-onset HF, improvement and persistence of HF symptoms, and worsening HF^7^. Despite novel advances in HF treatments, recent studies have revealed that the annual incidence of new-onset HF remains constant, with patients at a high risk of all-cause mortality and worsening HF^4,8^. Consequently, there is an anticipated increase in focus on the management of patients with new-onset HF.

While the uptake of guideline-directed medical therapy (GDMT) for patients with newly diagnosed HFrEF is often suboptimal in real-world settings^9^, and registries such as TITRATE-HF have demonstrated the feasibility of rapid GDMT optimization^10^, comprehensive evaluations encompassing all three LVEF subtypes, particularly in newly diagnosed HFmrEF and HFpEF patients, remain limited. Contemporary registries including the Swedish Heart Failure Registry^11^, ASIAN-HF^12^, and JROADHF^13^ have provided important insights into HF epidemiology and management; however, prior research has largely relied on cohorts including both incident and prevalent cases, as there are a limited number of cohorts following patients with new-onset HF in the general population in Japan^14^. Furthermore, the incidences of clinical events in newly diagnosed HF patients have not been fully established in contemporary clinical settings where newer treatment options were introduced. Herein, we investigated the patterns of pharmacological treatments and clinical events among newly diagnosed HF patients, stratified by LVEF, based on a nationwide hospital-based cohort in Japan.

## Methods

### Study design, data source, and study population

This study was a retrospective cohort study utilizing a Japanese hospital-based database provided by Medical Data Vision Co., Ltd. (MDV; Tokyo, Japan). The MDV database is a hospital administrative database collected from 485 acute hospitals in Japan, covering 44 million people in all age groups nationwide. As of October 2023, this database covers approximately 9.7% of the Japanese population and 26.6% of the acute-care hospitals. The database comprises health insurance claims data and clinical information recorded in the Diagnosis Procedure Combination reporting form^15^, including hospital diagnoses based on the International Classification of Diseases 10th revision, medical procedures, administration of in-hospital medications, prescriptions, and laboratory data^16^. The study period was from September 1, 2016 to July 31, 2024.

The study population was defined as newly diagnosed adults (aged ≥ 18 years) with HF, stratified by LVEF. Patients were eligible to enter the cohort on the date of their first HF diagnosis (i.e., index date), identified based on the diagnostic codes between January 1, 2020, and July 31, 2023. The LVEF was assessed within 90 days prior to or after the first HF diagnosis. We used the LVEF value recorded closest to the first HF diagnosis as the LVEF at index, considered in the assignment of LVEF subtype at diagnosis. In cases where two LVEF measurements were recorded equidistantly, before and after the first HF diagnosis, the first instance was considered. The study population represents patients newly diagnosed HF who were managed in acute-care hospital settings. Patients were excluded if they had less than 365 days of observability before the index date. This 365-day requirement was set to identify the incident HF cases by excluding prevalent HF cases recorded during this period and ascertaining the information of baseline comorbidities and prior medication use. In addition, patients with a history of heart transplant or durable ventricular assist device (VAD) prior to the index date were also excluded from this study. Patients were followed until censorship (death, heart transplant, or VAD use), disenrollment from the database, or the end of the study period, whichever came first.

### Variables and outcomes

Demographics, comorbidities, procedures, and comedications were collected during the 12 months prior to the index date. The Charlson Comorbidity Index was calculated based on a previously reported method^17^. A list of the variables used to assess the eligibility, comorbidity, and comedications of the study population can be found in Supplementary Table S1. The primary objective of this study was an evaluation of the patterns of pharmacological treatments in newly diagnosed HF patients. Clinical events were assessed in the secondary objectives. Prescriptions of HF medications (e.g., ACEis, ARBs, ARNI, MRAs, BBs, and SGLT2is) were assessed during the follow-up period. A combination regimen of HF medications was considered if an overlap of multiple stackable treatments (i.e., ACEi/ARB/ARNI, BB, MRA, and SGLT2i) continued for over 14 days. A treatment was considered to be continued if the gap period between two prescriptions was less than 30 days. These thresholds were determined based on the consideration of typical intervals in the Japanese hospital prescription systems and in alignment with prior pharmacoepidemiologic studies of HF treatment patterns using Japanese claims database^18^. During the follow-up period, the information on clinical events including in-hospital all-cause mortality, in-hospital cardiovascular death, hospitalization for HF (HHF), urgent HF visit (outpatient or emergency visits for HF with a record of intravenous diuretics, inotropes, or vasodilator use), and cardiovascular event (a composite event comprising HHF, non-fatal myocardial infarction, and non-fatal stroke) were collected. Because death records in the MDV database were limited to deaths occurring within the reporting hospitals, out-of-hospital deaths were not captured. Detailed definitions of clinical events are listed in Supplementary Table S2.

### Statistical analyses

Continuous variables were summarized using means with standard deviation (SD) or medians with first and third quartiles (Q1, Q3). Categorical variables were summarized using counts (n) and percentages (%). Missing data were not imputed. Prescription rates for the pharmacological treatment of HF (i.e., the cumulative numbers and percentages of patients receiving each HF medication class) were summarized at 6-, 12-, 18-, and 24-months of follow-up. Sankey diagrams were depicted to show the transitions of the pharmacological treatments over the first year after the index date. Furthermore, the association of clinical variables with the initiation of treatment with specific HF medication classes were assessed using Fine-Gray sub-distribution hazard models with death as a competing risk and the corresponding sub-distribution hazard ratios (HRs) of the treatment initiation with 95% confidence intervals (CIs), among treatment naïve patients with respective HF medication classes. For the clinical events, the number of patients with events and the incidence rates per 100 person-years were reported. Fine-Gray sub-distribution hazard models with death as a competing risk were employed to compute the HRs of clinical events in patients with HFmrEF and HFpEF compared with HFrEF. These analyses were intended to identify associations, not causal effects. Models were adjusted by demographic and clinical characteristics, as listed in Supplementary Table S3. To evaluate multi-collinearity, the variance inflation factor^19^ was calculated for each covariate. Covariates with a variance inflation factor exceeding 5 were excluded from the model. *P*-values < 0.05 were considered statistically significant. The analyses were performed in the overall study population and stratified by baseline LVEF category, i.e., HFrEF (LVEF < 40%), HFmrEF (LVEF 40–49%), HFpEF (LVEF ≥ 50%). All statistical analyses were conducted using the statistical software R version 4.0 or higher and SAS (SAS Institute, Inc; Cary, NC) version 9.4 or higher. Reporting of this study followed the STROBE (Strengthening the Reporting of Observational Studies in Epidemiology) statement^20^, as detailed in Supplementary Table S4.

### Ethics statement

This study was conducted in accordance with the Declaration of Helsinki. Informed consent was waived, since the study utilized only anonymized and de-identified data. The use of anonymized and de-identified data was in compliant to the Japan’s local regulations including Personal Information Protection Law and Ethical Guidelines for Medical and Biological Research Involving Human Subjects. The study protocol was reviewed and approved by an independent Ethics Committee in the specified nonprofit organization, MINS (formal name of organization; approval number 250203). An independent ethics committee of the Specified Non-profit Organization MINS waived the need for informed consent for the study based on the Japan’s regulations related to the use of anonymized and de-identified data.

## Results

We identified 44,337 patients aged ≥ 18 years with a first confirmed diagnosis of HF and with records of LVEF within 90 days prior to or after the first HF diagnosis. Among these individuals, 25,372 patients with insufficient observability prior to the index date and 2964 patients with a documented history of heart transplant or semi-permanent VAD were excluded. Finally, a total of 16001 (36.1%) patients were included in the analysis, among which 5473 (34.2%), 3053 (19.1%), and 7475 (46.7%) patients were categorized as having HFrEF, HFmrEF, and HFpEF, respectively. The mean (± SD) duration of the follow-up period was 301.1 ± 238.0 days.

### Clinical characteristics of the newly diagnosed HF patients

Table 1 shows the clinical characteristics in the overall population and stratified by LVEF category. Overall, the mean (± SD) age was 81.1 ± 11.8 years, while 48.2% were female. Patients with HFpEF were older (mean age: 82.9 years) and more often women (55.8%) than those with HFrEF (78.4 years; 38.8%) and HFmrEF (81.7 years; 46.4%). Commonly observed comorbidities included hypertension (61.3%), diabetes mellitus (36.8%), atrial fibrillation (33.4%), dyslipidemia (32.9%), and coronary artery disease (28.9%). Among the LVEF subtypes, HFpEF had the highest prevalence of atrial fibrillation (36.0%), anemia (23.0%), and valvular heart disease (18.1%), and the lowest prevalence of coronary artery disease (24.8%). In the overall population, 4128 (25.8%) patients were prescribed HF medications, including 2240 (14.0%), 1198 (7.5%), 553 (3.5%), and 354 (2.2%) patients on ACEi/ARB/ARNI, BB, MRA, and SGLT2i, respectively. There were no notable differences in baseline medications across the LVEF subtypes.

**Table 1:** Clinical characteristics in the overall population and stratified by left-ventricular ejection fraction category.

	Overall (N = 16,001)	HFrEF (N = 5473)	HFmrEF (N = 3053)	SMD^†^	HFpEF (N = 7475)	SMD^†^
Age (years)						
Mean ± SD	81.1 ± 11.8	78.4 ± 12.9	81.7 ± 11.0	0.28	82.9 ± 10.8	0.38
Median (Q1, Q3)	84 (75, 89)	81 (72, 88)	84 (76, 89)	–	85 (78, 90)	–
Age group (years), n (%)						
≥ 18–44	198 (1.2)	115 (2.1)	27 (0.9)	–0.10	56 (0.8)	–0.11
≥ 45–64	1,279 (8.0)	656 (12.0)	209 (6.9)	–0.18	414 (5.5)	–0.23
≥ 65–74	2,187 (13.7)	922 (16.9)	389 (12.7)	–0.12	876 (11.7)	–0.15
≥ 75	12,337 (77.1)	3,780 (69.1)	2428 (79.5)	0.24	6129 (82.0)	0.30
Gender, female, n (%)	7710 (48.2)	2125 (38.8)	1417 (46.4)	0.15	4168 (55.8)	0.34
Charlson comorbidity index						
Mean ± SD	4.0 ± 3.0	4.0 ± 3.0	4.1 ± 3.0	0.03	4.0 ± 3.0	0.00
Comorbidities, n (%)						
Hypertension	9800 (61.3)	3208 (58.6)	1885 (61.7)	0.06	4707 (63.0)	0.09
Diabetes mellitus	5891 (36.8)	2146 (39.2)	1198 (39.2)	0.00	2547 (34.1)	–0.11
Atrial fibrillation	5337 (33.4)	1632 (29.8)	1011 (33.1)	0.07	2694 (36.0)	0.13
Dyslipidemia	5271 (32.9)	1821 (33.3)	1021 (33.4)	0.00	2429 (32.5)	–0.02
Coronary artery disease	4617 (28.9)	1773 (32.4)	988 (32.4)	–0.00	1856 (24.8)	–0.17
Anemia	3343 (20.9)	951 (17.4)	676 (22.1)	0.12	1716 (23.0)	0.14
Chronic kidney disease	3222 (20.1)	1030 (18.8)	682 (22.3)	0.09	1510 (20.2)	0.03
Cerebrovascular disease	2617 (16.4)	873 (16.0)	495 (16.2)	0.00	1249 (16.7)	0.02
Valvular heart disease	2580 (16.1)	751 (13.7)	480 (15.7)	0.06	1349 (18.1)	0.12
Cancer	2478 (15.5)	866 (15.8)	463 (15.2)	–0.02	1149 (15.4)	–0.01
Dementia	1920 (12.0)	597 (10.9)	374 (12.3)	0.04	949 (12.7)	0.06
Myocardial infarction	1608 (10.1)	755 (13.8)	419 (13.7)	–0.00	434 (5.8)	–0.27
Obesity	951 (5.9)	268 (4.9)	180 (5.9)	0.04	503 (6.7)	0.08
Hyperkalemia	379 (2.4)	119 (2.2)	69 (2.3)	0.00	191 (2.6)	0.03
Baseline treatment, n (%)						
ACEi	343 (2.1)	120 (2.2)	84 (2.8)	0.04	139 (1.9)	–0.02
ARB	1851 (11.6)	481 (8.8)	388 (12.7)	0.13	982 (13.1)	0.14
ARNI	169 (1.1)	47 (0.9)	33 (1.1)	0.02	89 (1.2)	0.03
BB	1198 (7.5)	299 (5.5)	264 (8.7)	0.12	635 (8.5)	0.12
MRA	553 (3.5)	161 (2.9)	119 (3.9)	0.05	273 (3.7)	0.04
SGLT2i	354 (2.2)	134 (2.5)	77 (2.5)	0.00	143 (1.9)	–0.04
Oral diuretics	1500 (9.4)	394 (7.2)	310 (10.2)	0.11	796 (10.7)	0.12
Statins	1782 (11.1)	522 (9.5)	362 (11.9)	0.08	898 (12.0)	0.08
Lipid lowering drugs other than statins	221 (1.4)	50 (0.9)	51 (1.7)	0.07	120 (1.6)	0.06
Antiplatelet	1081 (6.4)	332 (6.1)	235 (7.7)	0.06	451 (6.0)	–0.00
Oral anticoagulants	1294 (8.1)	298 (5.4)	249 (8.2)	0.11	747 (10.0)	0.17
Calcium-channel blockers	2510 (15.7)	628 (11.5)	488 (16.0)	0.13	1394 (18.7)	0.20
Insulin	948 (5.9)	332 (6.1)	207 (6.8)	0.03	409 (5.5)	–0.03
Biguanides	453 (2.7)	186 (3.4)	82 (2.7)	–0.04	167 (2.2)	–0.07
DPP4i	1076 (6.7)	373 (6.8)	229 (7.5)	0.03	474 (6.3)	–0.02
GLP-1RA	150 (0.9)	50 (0.9)	29 (1.0)	0.00	71 (1.0)	0.00
Screening and diagnostic measures*, n (%)						
Echocardiography**	4518 (28.2)	1335 (24.4)	884 (29.0)	0.10	2299 (30.8)	0.14
Electrocardiogram	5062 (31.6)	1461 (26.7)	985 (32.3)	0.12	2616 (35.0)	0.18
NT-proBNP or BNP blood test	3256 (20.4)	886 (16.2)	646 (21.2)	0.13	1724 (23.1)	0.17
Hospitalization within 1 year, n (%)	3940 (24.6)	1224 (22.4)	790 (25.9)	0.08	1926 (25.8)	0.08
ED visit within the previous year, n (%)	2221 (13.9)	698 (12.8)	484 (15.9)	0.09	1039 (13.9)	0.03

*Recorded within 90 days prior to the index date. **The number of percentage of patients undergoing echocardiography within 90 days prior to the index date. On the other hand, LVEF records of patients included in the study were assessed within 90 days prior to or after the index date. ^†^Standardized mean difference with HFrEF. Abbreviations: ACEi, angiotensin-converting enzyme inhibitor; ARB, angiotensin receptor blocker; ARNI, angiotensin receptor blocker neprilysin inhibitor; BB, beta-blocker; BNP, brain natriuretic peptide; DPP4i, dipeptidyl peptidase-4 inhibitor; ED, emergency department; GLP-1RA, glucagon-like peptide-1 receptor agonist; HFrEF, heart failure with reduced ejection fraction; HFmrEF, heart failure with mildly reduced ejection fraction; HFpEF, heart failure with preserved ejection fraction; MRA, mineralocorticoid receptor antagonist; NT-proBNP, N-terminal pro-brain natriuretic peptide; SD, standard deviation; SGLT2i, sodium-glucose cotransporter-2 inhibitor; SMD, standardized mean difference.

### Patterns of pharmacological and other treatments after the first HF diagnosis

**Fig. 1:**
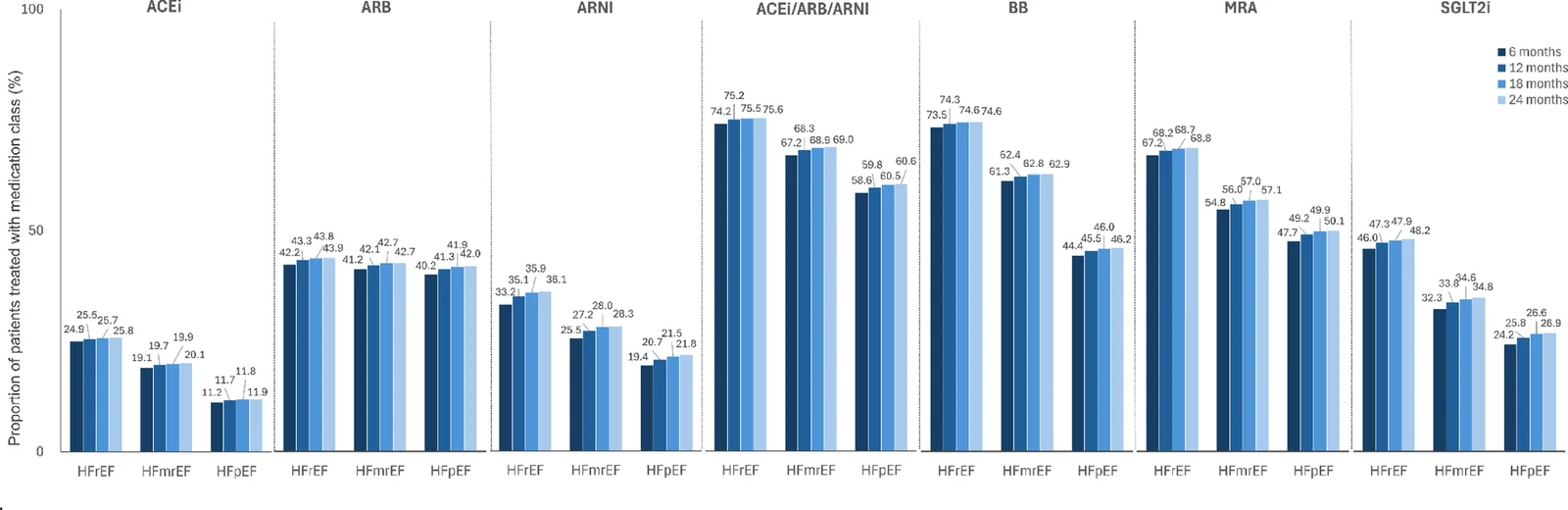
Prescription rates of pharmacological treatments after the first heart failure diagnosis, stratified by left-ventricular ejection fraction category. Prescription rates were calculated using the cumulative number of patients receiving each medication class at each time point divided by the total number of patients in each LVEF subtype. ACEi, angiotensin-converting enzyme inhibitor; ARB, angiotensin receptor blocker; ARNI, angiotensin receptor blocker neprilysin inhibitor; BB, beta-blocker; HFrEF, heart failure with reduced ejection fraction; HFmrEF, heart failure with mildly reduced ejection fraction; HFpEF, heart failure with preserved ejection fraction; LVEF, left-ventricular ejection fraction; MRA, mineralocorticoid receptor antagonist; SGLT2i, sodium-glucose cotransporter-2 inhibitor.

Within the first 6 months of follow-up, the prescription rates were 17.4% for ACEi, 41.1% for ARB, 25.3% for ARNI, 65.6% for pooled ACEi/ARB/ARNI, 57.6% for BB, 55.7% for MRA, and 33.2% for SGLT2i. Figure 1 shows the prescription rates of pharmacological treatments stratified by LVEF category. The proportion of patients treated with a medication class showed only a slight increase across all LVEF subtypes, from 6 to 12 months post-diagnosis and onward throughout the follow-up period. Prescription rates of all four medication classes were lower in the HFpEF subgroup compared to HFrEF and HFmrEF. This is expected for ACEi/ARB/ARNI, BB, and MRA, as these are primarily guideline-recommended for HFrEF, and their use in HFpEF is largely attributable to comorbidity management (e.g., hypertension, coronary artery disease, atrial fibrillation).

**Fig. 2:**
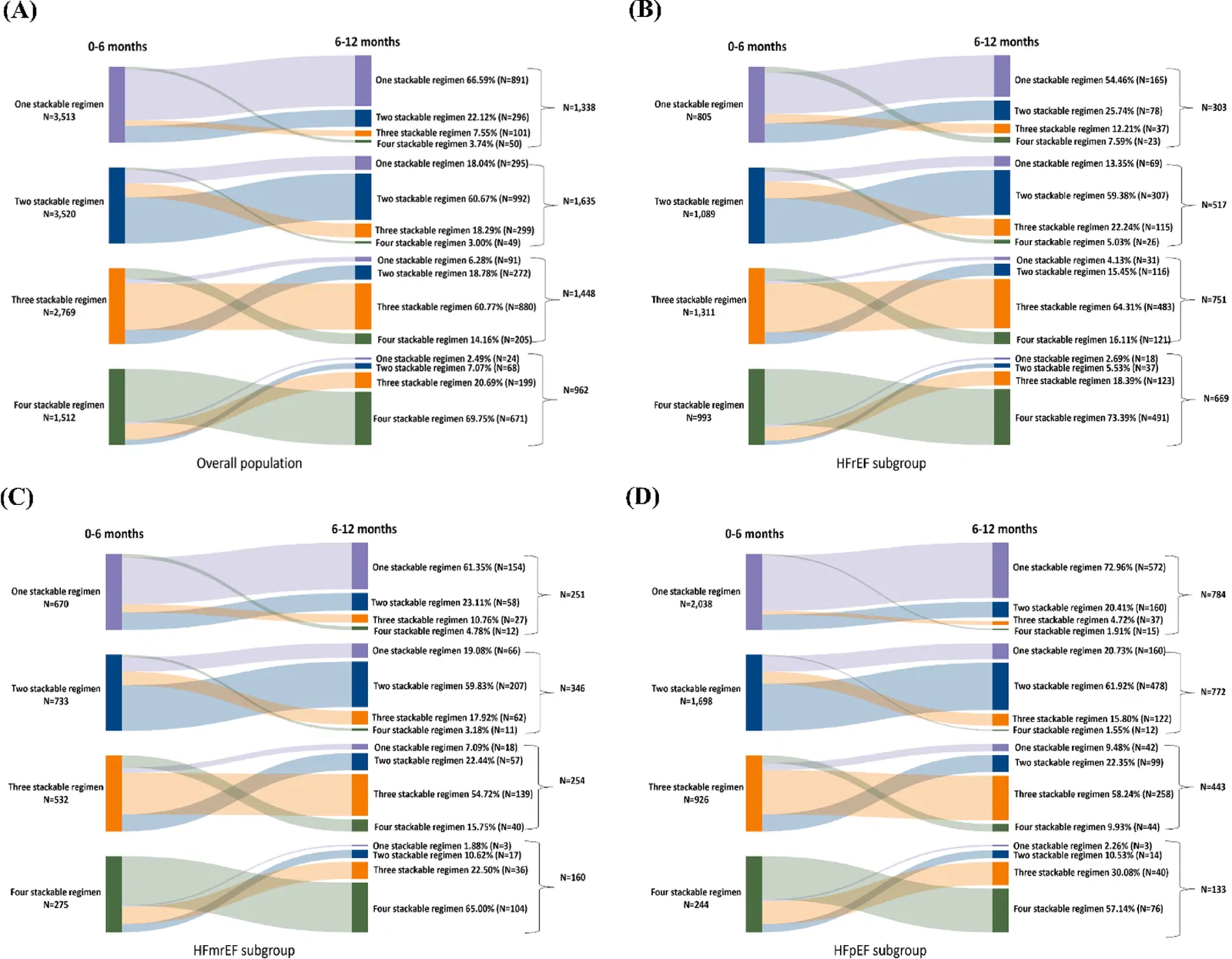
Sankey diagrams of the patterns of combination regimens of pharmacological treatments in the overall population and stratified left-ventricular ejection fraction categories. Panel (**A**) shows the results for the overall population, while Panels (**B**), (**C**), and (**D**) show the results for the HFrEF, HFmrEF, and HFpEF subgroups, respectively. The stackable regimen includes ACEi/ARB/ARNI, BB, MRA, and SGLT2i. Diuretics were not counted, for the number of combination regimens. ACEi, angiotensin-converting enzyme inhibitor; ARB, angiotensin receptor blocker; ARNI, angiotensin receptor blocker neprilysin inhibitor; BB, beta-blocker; HFrEF, heart failure with reduced ejection fraction; HFmrEF, heart failure with mildly reduced ejection fraction; HFpEF, heart failure with preserved ejection fraction; MRA, mineralocorticoid receptor antagonist; SGLT2i, sodium-glucose cotransporter-2 inhibitor.

Figure 2 shows Sankey diagrams of the patterns of combination regimens of pharmacological treatments overall population and stratified LVEF category. In the overall population, 3513 (22.0%), 3520 (22.0%), 2769 (17.3%), and 1512 (9.4%) patients were on mono-, dual-, triple-, and quadruple-therapy, respectively within 6 months. In the HFrEF subgroup, triple therapy was the most prevalent combination regimen, followed by dual therapy. In contrast, the HFmrEF subgroup predominantly received dual therapy, with monotherapy as the second most common regimen, while the HFpEF subgroup primarily utilized monotherapy. In the overall population, more than 60% of patients experienced no changes in the number of concomitant medication classes in 6–12 months. The most common changes included addition of one agent for those on mono- or dual-therapy (22.1% and 18.3%, respectively) and reduction of one agent for those on triple (18.8%) or quadruple therapy (20.7%). The detailed prescription rates of each combination regimen are reported in Supplementary Table S5. In the overall population, patients receiving each medication class demonstrated varying rates of treatment continuation (Supplementary Figure S1). The discontinuation rates of these medication classes varied from 22 to 38%, with ARB exhibiting the highest rate of discontinuation. However, the high ARB discontinuation rate likely reflects, at least in part, guideline concordant class switching from ARB to ARNI rather than true treatment cessation. Compared to the other subgroups, the HFrEF subgroup showed numerically lower discontinuation rates for each medication class.

**Fig. 3:**
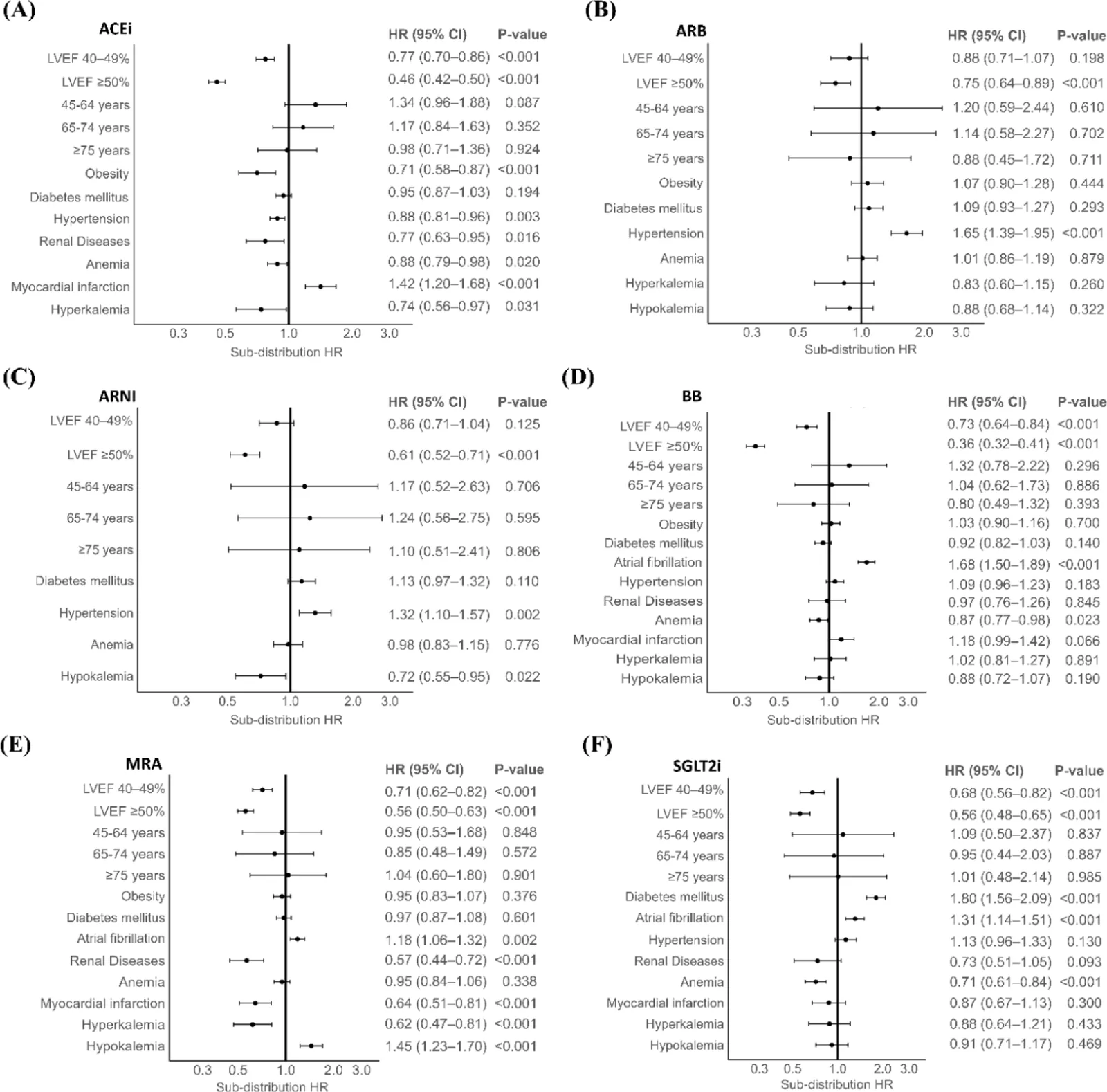
Associations between key clinical variables and the initiation of pharmacological treatments based on the Fine–Gray sub-distribution hazard models. Panels (**A**), (**B**), (**C**), (**D**), (**E**), and (**F**) show the results for ACEi, ARB, ARNI, BB, MRA, and SGLT-2i, respectively. For LVEF categories, LVEF < 40% was the reference group. For age categories, age group 18–44 was the reference group. Covariates with a variance inflation factor exceeding 5 were excluded from the model. All the variables used to adjust the multivariable models are listed in Supplementary Table S3. ACEi, angiotensin-converting enzyme inhibitor; ARB, angiotensin receptor blocker; ARNI, angiotensin receptor blocker neprilysin inhibitor; BB, beta-blocker; LVEF, left-ventricular ejection fraction; MRA, mineralocorticoid receptor antagonist; SGLT2i, sodium-glucose cotransporter-2 inhibitor.

Factors associated with initiation of each medication class included the baseline LVEF category, hypertension, obesity, diabetes mellitus, renal diseases, atrial fibrillation, myocardial infarction, hypokalemia, hyperkalemia, and anemia. Forest plots showing the sub-distribution hazard ratios of these clinical variables, estimated using Fine-Gray sub-distribution models are presented in Fig. 3. LVEF ≥ 50% was associated with significantly lower hazards of treatment initiation for all medication classes compared with LVEF < 40%. For ACEi/ARB/ARNI, BB, and MRA, this finding is consistent with current guideline recommendation, which do not provide Class I recommendations for these agents in HFpEF for HF management specifically, although they may be indicated for concurrent comorbidities. LVEF 40–49% was associated with significantly lower hazards of treatment initiation for ACEi, BB, MRA, and SGLT2i, compared with LVEF < 40%. With respect to the recommended treatments, the sub-distribution HR (95% CI) of ARNI initiation for LVEF 40–49% was 0.86 (0.71–1.04). The sub-distribution HRs (95% CI) of SGLT2i initiation for LVEF 40–49% and LVEF ≥ 50% were 0.68 (0.56–0.82) and 0.56 (0.48–0.65), respectively.

### Clinical events

Table 2 shows the clinical events in the overall population and stratified by LVEF category. In the overall population, the one-year incidence rates per 100 person-years (95% CI) of in-hospital all-cause mortality, in-hospital cardiovascular death, HHF, urgent HF visit, and cardiovascular events were 23.0 (22.1–24.0), 16.6 (15.8–17.4), 23.3 (22.3–24.2), 40.7 (39.4–42.0), and 25.4 (24.4–26.3), respectively. Corresponding numbers (%) of patients with each event within one year were 2275 (14.2%), 1641 (10.3%), 2299 (14.4%), 4018 (25.1%), and 2503 (15.6%), respectively. The occurrence of clinical events was mainly observed during the first 12 months, but increased continuously thereafter (Supplementary Figure S2). In unadjusted analyses, HFrEF patients exhibited numerically higher incidence rates for HHF, urgent HF visit, and cardiovascular events compared to HFmrEF and HFpEF patients, while in-hospital all-cause mortality rates were comparable across subtypes (Table 2). Table 3 presents the sub-distribution HRs of clinical events in each LVEF subtype. The risks of clinical events did not differ significantly, for all clinical events in patients with HFmrEF compared to HFrEF. In the HFpEF subgroup, lower risks of HHF, urgent HF visit, and cardiovascular events were observed than in the HFrEF subgroup, although the risks of in-hospital all-cause mortality and in-hospital cardiovascular death did not differ significantly.

**Table 2:** Clinical events in the overall study population and stratified by left-ventricular ejection fraction category.

	Overall	HFrEF	HFmrEF	HFpEF
	(N = 16,001)	(N = 5473)	(N = 3053)	(N = 7475)
In-hospital all-cause mortality				
Within 12 months				
Number of patients with event, n (%)	2275 (14.2)	803 (14.7)	433 (14.2)	1039 (13.9)
Incidence rate, per 100 person-years (95% CI)	23.0 (22.1–24.0)	23.3 (21.7–24.9)	23.3 (21.1–25.5)	22.7 (21.4–24.1)
Within 24 months				
Number of patients with event, n (%)	2546 (15.9)	892 (16.3)	485 (15.9)	1169 (15.6)
Incidence rate, per 100 person-years (95% CI)	19.5 (18.8–20.3)	19.4 (18.2–20.7)	19.6 (17.9–21.4)	19.6 (18.5–20.7)
In-hospital cardiovascular death				
Within 12 months				
Number of patients with event, n (%)	1641 (10.3)	621 (11.4)	325 (10.7)	695 (9.3)
Incidence rate, per 100 person-years (95% CI)	16.6 (15.8–17.4)	18.0 (16.6–19.4)	17.5 (15.6–19.4)	15.2 (14.1–16.3)
Within 24 months				
Number of patients with event, n (%)	1745 (10.9)	649 (11.9)	347 (11.4)	749 (10.0)
Incidence rate, per 100 person-years (95% CI)	13.4 (12.8–14.0)	14.1 (13.1–15.2)	14.0 (12.6–15.5)	12.6 (11.7–13.5)
HHF				
Within 12 months				
Number of patients with event, n (%)	2299 (14.4)	855 (15.6)	442 (14.5)	1002 (13.4)
1 HHF*	1949 (12.2)	720 (13.2)	370 (12.1)	859 (11.5)
2 HHF*	266 (1.7)	101 (1.9)	50 (1.6)	115 (1.5)
3 HHF*	71 (0.4)	29 (0.5)	18 (0.6)	24 (0.3)
> 3 HHF*	13 (0.1)	5 (0.1)	4 (0.1)	4 (0.1)
≥ 1 readmission within 30 days**	145 (0.9)	62 (1.1)	26 (0.9)	57 (0.8)
Incidence rate, per 100 person-years (95% CI)	23.3 (22.3–24.2)	24.8 (23.1–26.5)	23.8 (21.6–26.0)	21.9 (20.6–23.3)
Within 24 months				
Number of patients with event, n (%)	2552 (16.0)	926 (16.9)	496 (16.3)	1130 (15.1)
Incidence rate, per 100 person-years (95% CI)	19.6 (18.8–20.3)	20.2 (18.9–21.5)	20.1 (18.3–21.8)	18.9 (17.8–20.0)
Urgent HF visit				
Within 12 months				
Number of patients with event, n (%)	4018 (25.1)	1489 (27.2)	795 (26.0)	1734 (23.2)
1 visit*	1626 (10.2)	594 (10.9)	313 (10.3)	719 (9.6)
2 visits*	498 (3.1)	187 (3.4)	98 (3.2)	213 (2.9)
3 visits*	316 (2.0)	119 (2.2)	63 (2.1)	134 (1.8)
> 3 visits*	1578 (9.9)	589 (10.8)	321 (10.5)	668 (8.9)
Incidence rate, per 100 person-years (95% CI)	40.7 (39.4–42.0)	43.2 (41.0–45.4)	42.8 (39.9–45.8)	38.0 (36.2–39.7)
Within 24 months				
Number of patients with event, n (%)	4480 (28.0)	1638 (29.9)	876 (28.7)	1966 (26.3)
Incidence rate, per 100 person-years (95% CI)	34.4 (33.4–35.4)	35.7 (34.0–37.4)	35.4 (33.1–37.8)	32.9 (31.5–34.4)
Cardiovascular event***				
Within 12 months				
Number of patients with event, n (%)	2503 (15.6)	935 (17.1)	504 (16.5)	1064 (14.2)
Incidence rate, per 100 person-years (95% CI)	25.4 (24.4–26.3)	27.1 (25.4–28.9)	27.2 (24.8–29.5)	23.3 (21.9–24.7)
Within 24 months				
Number of patients with event, n (%)	2768 (17.3)	1012 (18.5)	558 (18.3)	1198 (16.0)
Incidence rate, per 100 person-years (95% CI)	21.2 (20.5–22.0)	22.1 (20.7–23.4)	22.6 (20.7–24.4)	20.1 (18.9–21.2)

*Number of events observed within 12 months. **Patients with at least one readmission within 30 days after a prior HHF. ***A composite event comprising HHF, non-fatal myocardial infarction, and non-fatal stroke. CI, confidence interval; HF, heart failure; HHF, hospitalization for heart failure; HFrEF, heart failure with reduced ejection fraction; HFmrEF, heart failure with mildly reduced ejection fraction; HFpEF, heart failure with preserved ejection fraction.

**Table 3:** Sub-distribution hazard ratios of clinical events in each left-ventricular ejection fraction subgroup based on Fine–Gray sub-distribution hazard models.

	Sub-distribution HR (95% CI)	*P*-value
In-hospital all-cause mortality		
HFrEF	Reference	–
HFmrEF	0.93 (0.74–1.16)	0.519
HFpEF	0.91 (0.76–1.08)	0.281
In-hospital cardiovascular death		
HFrEF	Reference	–
HFmrEF	0.94 (0.70–1.26)	0.694
HFpEF	0.86 (0.68–1.09)	0.218
HHF		
HFrEF	Reference	–
HFmrEF	0.91 (0.81–1.01)	0.085
HFpEF	0.82 (0.75–0.89)	< 0.001
Urgent HF visit*		
HFrEF	Reference	–
HFmrEF	0.97 (0.82–1.16)	0.754
HFpEF	0.79 (0.69–0.92)	0.002
Cardiovascular event*		
HFrEF	Reference	–
HFmrEF	0.98 (0.88–1.09)	0.736
HFpEF	0.91 (0.83–0.99)	0.035

All the variables used to adjust the multivariable models are listed in Supplementary Table S3. The model was applied to the data collected from entire follow-up periods in each patient. *A composite event comprising HHF, non-fatal myocardial infarction, and non-fatal stroke. CI, confidence interval; HF, heart failure; HR, hazard ratio; HHF, hospitalization for heart failure; HFrEF, heart failure with reduced ejection fraction; HFmrEF, heart failure with mildly reduced ejection fraction; HFpEF, heart failure with preserved ejection fraction.

## Discussion

This large contemporary cohort study describes the longitudinal patterns of pharmacological treatment initiations and clinical events in newly diagnosed HF patients across LVEF subtypes in real-world settings in Japan. The study period (2020–2024) spans the critical window during SGLT2i become available for HF across LVEF subtypes, providing a timely baseline for evaluating the impact of the policy and guideline revisions in clinical practice.

The study findings underscore the critical importance of initiating HF treatment promptly after diagnosis and optimizing the treatment regimen for patients. Newly diagnosed HF patients represent the pivotal first decline in the trajectory of HF progression^21^. In this study, the mean age of the overall population was 81 years, reflecting the incidence of HF in Japan’s aging society as reported in a recent population-based study^22^. The LVEF subtype distribution in the present study (HFrEF 34.2%, HFmrEF 19.1%, HFpEF 46.7%) is consistent with previously reported distributions (HFrEF 37.4%, HFmrEF 17.5%, HFpEF 45.1%) in the JROADHF study, supporting the representativeness of this cohort^13^. The predominance of HFpEF in the present study is consistent with the well-documented epidemiological trend of increasing HFpEF prevalence, particularly in elderly aging population^23^. Patients had high prevalences of comorbidities (e.g., hypertension, diabetes mellitus, chronic kidney disease, and coronary artery disease). In the present study, prescription rates within 6 months in HFrEF patients were: ACEi/ARB/ARNI 74.2%, BB 73.5%, MRA 67.2%, and SGLT2i 46.0%. Only 23.7% of HFrEF patients were on quadruple therapy at 6 months, indicating that the majority of HFrEF patients had not received the full guideline-recommended regimen. In HFpEF patients, the low prescription rates of ACEi/ARB, BB, and MRA may reflect guideline-concordant practice rather than treatment gaps; however, the prescription rates of SGLT2i were relatively low in HFmrEF (32.3%) and HFpEF (24.2%) patients. These findings suggest that the initiation of pharmacological treatment in newly diagnosed HF patients is suboptimal after the HF diagnosis.

After initiation, the maintenance of pharmacological therapy is equally important for optimal HF management. In our study, once a medication class was initiated, the continuation rates varied substantially across classes. The HFrEF subgroup showed numerically lower discontinuation rates compared to HFmrEF and HFpEF, consistent with stronger guideline recommendations to sustain the therapy in HFrEF. Several factors may influence medication persistence in this population, including side effects (e.g., hypotension, hyperkalemia, renal dysfunction), transition of care between acute and non-acute settings, polypharmacy burden in elderly patients, and the absence of clear guideline recommendations for certain medication classes in HFpEF. Dose optimization and titration is also a key component of GDMT, and our analysis of prescription rates may represent a further underestimation of treatment gap, as many patients who are prescribed GDMT may not reach target doses. In fact, suboptimal dose titrations of HF medications in clinical practice were reported in several studies^24,25^. Due to the methodological complexity and limitations in availability of data, the extent to which doses were up-titrated toward target levels during the post-6-month period could not be assessed in the present analysis. Future studies utilizing detailed prescription dose data should evaluate dose titration trajectories and attainment of maximally tolerated doses in this population. The observed treatment de-escalation in the present study may have several reasons such as documented side effects, adverse drug reactions, or clinical decision-making based on LVEF improvement. While some degree of medication adjustment may be clinically appropriate (e.g., in cases of intolerance or LVEF recovery), the observation that de-escalation occurs relatively frequently, particularly non-HFrEF patients, indicates concerns of potential premature discontinuation.

Previous studies demonstrated the benefits of early intervention and high-intensity care for HF patients^26,27^. The STRONG-HF trial tested a high-intensity care for acute HF patients to achieve GDMT early, resulting in improvements in the prognosis of the patients^27^. This study also showed the potential role of multidisciplinary teams in facilitating timely treatment initiation and optimization based on a structured high-intensity care protocol. However, it is also important to recognize that not all patients can be uniformly eligible for an intensive treatment. In the secondary analysis of the STRONG-HF trial, patients with low blood pressure and more congestion were less likely to be up-titrated to optimal GDMT doses early^28^. Consequently, some patients may not exhibit significant symptoms while treatment intensification continues to be under-optimized, leading to passive monitoring until deterioration of HF necessitates hospitalization. A recent study in the United States showed that a key barrier to prescribing quadruple GDMT in patients with HFrEF was clinical inertia, with physicians perceiving patients as clinically stable and without need for a particular medication^29^. Clinical inertia may be particularly pronounced in the context of newly diagnosed HF in elderly patients, where physicians may adopt a conservative approach due to concern about polypharmacy, falls, renal function decline, and hypotension risk. The present study shows only modest treatment optimization over the first six months and thereafter, underscoring the critical need for timely intervention in the early stage of HF management.

This study also showed that the prognosis of newly diagnosed HF patients, irrespective of LVEF subtype, remains poor in contemporary clinical settings. The similar mortality across LVEF subtypes, after adjustment for clinical characteristics, suggests that comorbidity burden–including hypertension, diabetes mellitus, chronic kidney disease, atrial fibrillation, and anemia–may be a more important determinant of prognosis than LVEF classification alone. Recent clustering and phenomapping studies have identified HF subgroups across LVEF boundaries, suggesting that LVEF-based classification may not fully capture the biological complexity of HF^30,31^. These findings support an approach to HF management that consider totality of patient characteristics rather than relying solely on LVEF-based treatment algorithms, consistent with the evolving understanding of HF as a heterogenous syndrome. The findings for the one-year incidence of in-hospital all-cause mortality (14.2%) and in-hospital cardiovascular death, which accounted for a large portion of in-hospital all-cause mortality irrespective of LVEF subtype, are comparable with the findings from previous reports based on real-world cohorts including de novo acute decompensated HF^14,32^, suggesting that the prognosis for HF patients has not improved over the past decade although cautious interpretation is required when comparing in-hospital mortality rates across studies. Patients with HFrEF exhibited a relatively high severity and cardiovascular risks, as indicated by numerically higher incidence rates of clinical events except in-hospital all-cause mortality. In contrast to findings from international registries that report lower cardiovascular mortality in HFpEF relative to HFrEF, our study found no significant difference in either in-hospital all-cause mortality or in-hospital cardiovascular death across LVEF subtypes. This discrepancy is likely attributable to the restriction of mortality ascertainment to in-hospital deaths, which inherently enriches for cardiovascular causes and may under-capture non-cardiovascular death that occur in non-acute settings. Furthermore, the elderly age of our cohort and the focus on newly diagnosed patients may contribute to a higher acute cardiovascular mortality risk in HFpEF patients compared to populations studied in other registries.

This study also highlighted a disparity in treatment initiations across different LVEF subtypes. The TITRATE-HF study demonstrated that rapid initiation of GDMT for HFrEF is feasible in clinical practice^33^. While the guideline recommendations and available treatment options allow a rigorous treatment approach for HFrEF, a more lenient approach tends to be taken when patients with HFpEF are admitted for the first time. Fewer recommended treatment options compared to HFrEF may be a part of reasons for low implementation rates of pharmacological treatments in HFmrEF and HFpEF patients. For instance, the low prescription rates in HFpEF patients should not automatically be interpreted as under-treatment, given that during the study period (2020–2024), Class I recommendations for HFpEF were limited. For medication classes without Class I recommendation in HFpEF (e.g., ACEi/ARB, BB, MRA), non-prescription may reflect guideline-concordant clinical practice rather than treatment gaps. The low prescription rates may also reflect appropriate clinical decisions driven by contraindications, intolerance, comorbidities, renal impairment, hypotension risk, or polypharmacy concerns. The high prevalence of hypertension (63.0% in HFpEF), atrial fibrillation (36.0% in HFpEF), and diabetes mellitus may explain much of the observed use of these agents in HFpEF patients. Nevertheless, the prescription rates for the recommended treatments such as ARNI for HFmrEF and SGLT2i for HFmrEF and HFpEF remained comparatively low in HFmrEF and HFpEF patients in the present study. Among these agents, SGLT2is carry the strongest evidence across all LVEF subtypes. The low prescription rates of SGLT2i may also be interpreted with several specific barriers to the uptake of SGLT2i use such as cost, access, and prescribers’ familiarity. Snowden, et al*.* reported several factors that may influence the management of HFpEF such as difficulties with diagnosis, unclear illness perceptions, and management disparities^34^. This study also reported that a sense of problem was conveyed in some patients and clinicians in the diagnostic process, complicated by non-specific symptoms, comorbidities, and variability in service provision. This raises the possibility that factors on the healthcare provider’s side may also play a role. The population of patients with HFpEF is diverse^35^, and the multidisciplinary team approach appears to be needed to address the lack of treatment implementation.

Collectively, this study underscores the importance of properly implementing the evidence-based therapies within each LVEF subtypes. The 2025 revision of the JCS/JHFS Guidelines on Diagnosis and Treatment of HF introduced SGLT2is, classified as a Class I recommendation for all LVEF categories, and ARNI as Class I for HFrEF and Class IIa for HFmrEF. Furthermore, Finerenone was classified as Class IIa for HFmrEF and HFpEF based on recent clinical trial results^7,36^. Our study period (2020–2024) predates the 2025 guideline update, and that the low prescription rates of SGLT2 inhibitors observed in HFmrEF and HFpEF should be interpreted in the context of evolving evidence base during the study period. The low overall prescription rates of SGLT2i and ARNI observed in this study reflect, in part, the recent market introduction of these agents during the study period. In fact, our exploratory analysis of prescription rates of SGLT2i and ARNI in the annual cross-sectional cohorts in each calendar year showed increasing trends in prescription rates of both SGLT2i and ARNI, towards the recent time periods (Supplementary Table S6). The emergence of novel HF medications and corresponding guideline updates are expected to substantially change prescribing patterns in future cohorts. It is essential to evaluate whether this enhancement can lead to improvements in the treatment patterns, outcomes, and prognosis of the patients. A re-evaluation in this context should be conducted in the coming years, to assess whether these advancements have indeed resulted in better patient outcomes.

This study has several limitations. First, the study population was identified based on hospital administrative data; therefore, the data were not collected for research purposes. Potential confounders such as New York Heart Association functional class and symptom severity, detail hemodynamic parameters, socioeconomic status and health literacy, and frailty indices were not captured in the database and thus not considered in the statistical models. The ascertainment of comorbidities based on diagnostic codes may underestimate the burden of certain comorbidities such as coronary artery disease, particularly subclinical disease, in this elderly population. In fact, the JCARE-CARD and CHART studies reported the prevalence of ischemic etiology in approximately 26–32% among enrolled HF patients while more recent CHART-2 study reported a higher proportion of ischemic heart disease^37–39^. LVEF data was collected in a 90-day pre/post window of initial HF diagnosis, as such the potential for temporal variability in LVEF to introduce misclassification of LVEF subtypes may not be fully denied. The dataset contains information on patients who have sought and received medical care in acute-care hospitals, and as a result, any patients with mild symptoms or patients treated by general practitioners may not be included in this study. The clinical profile of this population (e.g., higher acuity, higher comorbidity burden) may differ from HF patients diagnosed and managed entirely in primary care. Second, the information on care provided in medical facilities other than the hospitals covered by the MDV database were not recorded. Therefore, the possibility of missing drug prescriptions and other treatment records cannot be fully denied, in cases where a patient was transferred to another healthcare provider during the follow-up period. This may lead to underestimation of prescription rates, particularly for patients who were subsequently managed in outpatient or primary care settings. The magnitude of underestimation may differ across LVEF subtypes if referral and follow-up patterns vary. The 365-day observability requirement may systematically exclude patients with more transient hospital contacts, and that these patients may differ in disease severity and treatment exposure. The 365-day washout period for patient inclusion may reduce but does not eliminate the possibility of including prevalent HF cases, particularly for patients who received their initial HF diagnosis at a hospital not covered by MDV. Third, death records were available only in cases where patients died in the hospital, which may have led to an underestimation of the mortality rate in this study. Forth, the relatively short follow-up time may have led to an under-reporting of the clinical events and medication use occurring in longer-term follow-up. Even given these limitations, this study provides the critical aspects of treatment optimizations and outcomes associated with newly diagnosed HF across LVEF subtypes, based on comprehensive and representative data, especially within a short to medium term after the first diagnosis. It is also important to note that temporal changes in guideline recommendations and drug approvals during the study period may have influenced prescribing patterns, particularly for SGLT2i in HFmrEF and HFpEF patients. Finally, because this study is descriptive in nature, the findings from this study cannot establish causal relationships between treatment initiation and clinical outcomes.

## Conclusion

Based on a large contemporary cohort, we have reported comprehensive epidemiologic evaluations of newly diagnosed HF patients, including the initiation of pharmacological treatments and clinical events across LVEF subtypes in real-world settings in Japan. The findings underscore the suboptimal uptake of pharmacological treatment despite the poor prognosis of newly diagnosed HF patients in contemporary clinical settings. The disparities in initiations of recommended treatments reaffirm the importance of properly implementing the evidence-based therapies within each LVEF subtype.

## Supplementary Information

Below is the link to the electronic supplementary material.

Supplementary Material 1

## Data Availability

All necessary data required to interpret and conclude the findings of this study were included in the main text and supplementary materials.
