# Supplementary material for: Pharmacological treatments and clinical events in newly diagnosed heart failure patients stratified by ejection fraction in Japan
Source: Sci Rep. 2026 Jun 13;16:26919. doi: 10.1038/s41598-026-58004-0 (PMC13518946; doi:10.1038/s41598-026-58004-0)
Supplement: Supplementary file 1 [file 41598_2026_58004_MOESM1_ESM.pdf]

# SUPPLEMENTARY MATERIALS

**Table S1. List of variables used to assess eligibility, comorbidities, and medications in this study**……………2
**Table S2. Definitions of clinical events collected in this study**……………………………………………………6
**Table S3. List of clinical variables included in the multivariable models**…………….……………………………7
**Table S4. STROBE Checklist of the study**……………………………………………….……………………11
**Table S5. Combination therapy regimen within 12 months after the first heart failure diagnosis in overall population and stratified the left-ventricular ejection fraction category**…….…………….…………………..13
**Table S6. Prescription rates of SGLT2i and ARNI in the annual cross-sectional cohorts of HFmrEF/pEF in each calendar year between 2020 and 2024**…………………………………………………………………...14
**Figure S1. Sankey diagrams of the patterns of treatment continuations of pharmacological treatments in the overall population and stratified by left-ventricular ejection fraction category**….……………………………15
**Figure S2. Cumulative incidence curves of clinical events in patients stratified by left-ventricular ejection fraction categories**………..………………………….…………………………….….………………....19

## Supplementary Tables and Figures

**Table S1. List of variables used to assess eligibility, comorbidities, and medications in this study.**
**Abbreviations:** ACEi, angiotensin-converting-enzyme inhibitor; ARB, angiotensin-receptor blocker; ARNI, angiotensin-receptor blocker neprilysin inhibitor; ATC, anatomical therapeutic chemical; BB, beta-blocker; DPP4i, dipeptidyl peptidase-4 inhibitor; BNP, brain natriuretic peptide; GLP-1RA, glucagon-like peptide-1 receptor agonist; HF, heart failure; HFrEF, heart failure with reduced ejection fraction; HFmrEF, heart failure with mildly reduced ejection fraction; HFpEF, heart failure with preserved ejection fraction; ICD-10, International Classification of Diseases–10$^{th}$ edition; LVEF, left-ventricular ejection fraction; MRA, mineralocorticoid receptor antagonist; NT-proBNP, N-terminal pro-brain natriuretic peptide; SGLT2i, sodium-glucose cotransporter-2 inhibitor; and VAD, ventricular assist device.

| Variables | Definition |
|---|---|
| **Variables to assess eligibility** | |
| Age | Age at the time of first HF diagnosis |
| HF | A diagnostic code of HF (ICD-10, I11.0, I50.0, I50.1, I50.9) |
| HFrEF | Diagnosis of HF + LVEF <40% recorded within 90 days prior to or after the index date |
| HFmrEF | Diagnosis of HF + 40%≤ LVEF <50% recorded within 90 days prior to or after the index date |
| HFpEF | Diagnosis of HF + LVEF ≥50% recorded within 90 days prior to or after the index date |
| Heart transplant | Assessed based on receipt codes for heart transplant (150322310, 150322410, 150416270, 150322510, 150322610, 150416370, 150387710). |
| Semi-permanent VAD | Assessed based on receipt codes for semi-permanent VAD (150360110, 150360210, 150360310, 150360410). |
| **Comorbidities** | |
| Obesity | Obesity was defined based on diagnostic codes (ICD-10: E66.1, E66.2, E66.8, E66.9) or a body mass index ≥25 kg/m$^2$ recorded during one year prior to the index date. |
| Diabetes mellitus | Diabetes mellitus was defined based on diagnostic codes (ICD-10: E10, E10.0, E10.1, E10.2, E10.3, E10.4, E10.5, E10.6, E10.7, E10.9, E11, E11.0, E11.1, E11.2, E11.3, E11.4, E11.5, E11.6, E11.7, E11.9, E12, E13, E13.0, E13.1, E13.2, E13.3, E13.4, E13.5, E13.6, E13.7, E13.9, E14, E14.0, E14.1, E14.2, E14.3, E14.4, E14.5, E14.6, E14.9), or at least one prescription of a diabetes-related medication during one year prior to the index date. |
| Chronic kidney disease | Chronic kidney disease was defined based on diagnostic codes (ICD-10: N18.1, N18.2, N18.3, N18.4, N18.5) or two chronic kidney disease qualifying events ≥90 days apart (qualifying events are defined as clinical lab values indicating 25≤ estimated glomerular filtration rate <90 mL/min/1.73 m$^2$). |
| Coronary artery disease | Coronary artery disease was defined based on diagnostic codes (ICD-10: I20.0, I20.1, I20.8, I20.9, I21.0, I21.1, I21.2, I21.3, I21.4, I21.9, I22.0, I22.1, I22.8, I22.9, I23.0, I23.1, I23.2, I23.3, I23.4, I23.5, I23.6, I23.8, I24.0, I24.1, I24.8, I24.9, I25.1, I25.2, I25.3, I25.4, I25.5, I25.6, I25.8, I25.9) recorded during one year prior to the index date. |
| Atrial fibrillation | Atrial fibrillation was defined based on diagnostic codes (ICD-10: I48.0, I48.1, I48.2, I48.9) recorded during one year prior to the index date. |

| | |
|---|---|
| Hypertension | Hypertension was defined based on diagnostic codes (ICD-10: I10, I11.0, I11.9, I12.0, I12.9, I13.9, I15.0, I15.1, I15.2, I15.8, I15.9, I67.4) recorded within one year prior to the index date. |
| Dyslipidemia | Dyslipidemia was defined based on diagnostic codes (ICD-10: E78.0, E78.1, E78.2, E78.3, E78.4, E78.5, E78.6, E78.8, E78.9) recorded within one year prior to the index date. |
| Cerebrovascular disease | Cerebrovascular disease was defined based on diagnostic codes (ICD-10: I60.0, I60.1, I60.2, I60.3, I60.4, I60.5, I60.6, I60.7, I60.8, I60.9, I61.0, I61.1, I61.3, I61.4, I61.5, I61.6, I61.8, I61.9, I62.0, I62.1, I62.9, I63.0, I63.1, I63.2, I63.3, I63.4, I676, I63.5, I63.6, I63.8, I63.9, I64, I65.0, I65.1, I65.2, I65.3, I66.0, I66.1, I66.2, I66.3, I66.8, I66.9, I67.0, I67.1, I67.2, I67.3, I67.4, I67.5, I67.7, I67.8, I67.9, I69.0, I69.1, I69.3, I69.4) recorded within one year prior to the index date. |
| Anemia | Anemia was defined based on diagnostic codes (ICD-10: D50.0, D50.1, D50.8, D50.9, D51.0, D51.1, D51.2, D51.3, D51.8, D51.9, D52.0, D52.1, D52.8, D52.9, D53.0, D53.1, D53.2, D53.8, D53.9, D55.0, D55.1, D55.2, D55.9, D56.0, D56.1, D56.2, D56.3, D56.4, D56.9, D57.0, D57.1, D57.2, D57.3, D57.8, D58.0, D58.1, D58.2, D58.8, D58.9, D59.0, D59.1, D59.2, D59.3, D59.4, D59.5, D59.6, D59.9, D60.0, D60.9, D61.0, D60.0, D60.1, D61.1, D61.2, D61.3, D61.9, D62, D64.0, D64.1, D64.2, D64.3, D64.4, D64.8, D64.9), or a prescription of intravenous iron or erythropoietin stimulating agents within one year prior to the index date. |
| Valvular heart disease | Valvular heart disease was defined based on diagnostic codes (ICD-10: I05.0, I05.1, I05.2, I05.8, I05.9, I06.0, I06.1, I06.2, I06.9, I07.0, I07.1, I07.2, I07.8, I07.9, I08.0, I08.1, I08.2, I08.3, I08.8, I08.9, I34.0, I34.1, I34.2, I34.8, I35.0, I35.1, I35.2, I35.8, I35.9, I36.0, I36.1, I36.2, I36.9, I37.0, I37.1, I37.2, I37.9, I38, Q22.0, Q22.1, Q22.2, Q22.3, Q22.4, Q22.5, Q22.6, Q22.8, Q23.0, Q23.1, Q23.2, Q23.3, Q23.4, Q23.8) recorded within one year prior to the index date. |
| Myocardial infarction | Myocardial infarction was defined based on diagnostic codes (ICD-10: I21.0, I21.1, I21.2, I21.3, I21.4, I21.9, I22.0, I22.1, I22.8, I22.9, I23.0, I23.1, I23.2, I23.3, I23.4, I23.5, I23.6, I23.8, I25.2) recorded within one year prior to the index date. |
| Dementia | Dementia was defined based on diagnostic codes (ICD-10: F01.0, F01.1, F01.2, F01.9, F03, F05.1, G10, G20, G30.0, G30.1, G30.8, G30.9, G31.0, G31.1, G31.8) recorded within one year prior to the index date. |
| Hyperkalemia | Hyperkalemia was defined based on diagnostic code (ICD-10: E87.5), prescription of drugs to treat hyperkalemia (intravenous calcium chloride or calcium gluconate, hypertonic saline, insulin and glucose, beta agonists, intravenous sodium bicarbonate, or potassium binders), or laboratory data (serum potassium >5.5 mmol/L) within one year prior to the index date. |
| Cancer | Cancer was defined based on diagnostic codes (ICD-10: C00.0, C00.1, C00.2, C00.3, C00.4, C00.6, C00.8, C00.9, C01, C02.0, C02.1, C02.2, C02.9, C03.0, C03.1, C03.9, C04.0, C04.1, C04.9, C05.0, C05.1, C05.2, C05.9, C06.0, C06.1, C06.2, C06.9, C07, C08.0, C08.1, C08.9, C09.0, C09.1, C09.9, C10.0, C10.1, C10.2, C10.3, C10.4, C10.9, C11.0, C11.1, C11.2, C11.3, C11.9, C12, C13.0, C13.1, C13.2, C13.9, C14.0, C15.0, C15.1, C15.2, C15.3, C15.4, C15.5, C15.8, C15.9, C16.0, C16.1, C16.2, C16.3, C16.4, C16.5, C16.6, C16.9, C17.0, C17.1, C17.2, C17.9, C18.0, C18.1, C18.2, C18.3, C18.4, C18.5, C18.6, C18.7, C18.9, C19, C20, C21.0, C21.1, C22.0, C22.1, C22.2, C22.3, C22.4, C22.7, C22.9, C23, C24.0, C24.1, C24.8, C24.9, C25.0, C25.1, C25.2, C25.3, C25.4, C25.7, C25.8, |

| | |
|---|---|
| | C25.9, C26.1, C26.9, C30.0, C30.1, C31.0, C31.1, C31.2, C31.3, C31.9, C32.0, C32.1, C32.2, C32.3, C32.9, C33, C34.0, C34.1, C34.2, C34.3, C34.8, C34.9, C37, C38.0, C38.1, C38.2, C38.3, C38.4, C40.0, C40.1, C40.2, C40.3, C41.0, C41.1, C41.2, C41.3, C41.4, C41.9, C43.0, C43.1, C43.2, C43.3, C43.4, C43.5, C43.6, C43.7, C43.8, C43.9, C45.0, C45.1, C45.2, C45.9, C46.9, C47.0, C47.1, C47.2, C47.3, C47.4, C47.5, C47.6, C47.9, C48.0, C48.1, C48.2, C49.0, C49.1, C49.2, C49.3, C49.4, C49.5, C49.6, C49.9, C50.0, C50.1, C50.1, C50.2, C50.3, C50.4, C50.5, C50.6, C50.8, C50.9, C51.0, C51.1, C51.2, C51.9, C52, C53.0, C53.1, C53.8, C53.9, C54.0, C54.1, C54.2, C54.3, C54.9, C55, C56, C57.0, C57.9, C58, C60.0, C60.1, C60.2, C60.9, C61, C62.0, C62.1, C62.9, C63.0, C63.1, C63.2, C63.7, C63.9, C64, C65, C66, C67.0, C67.1, C67.2, C67.3, C67.4, C67.5, C67.6, C67.7, C67.9, C68.0, C68.1, C69.0, C69.1, C69.2, C69.3, C69.4, C69.5, C69.6, C70.0, C70.1, C71.0, C71.1, C71.2, C71.3, C71.4, C71.5, C71.6, C71.7, C71.8, C71.9, C72.0, C72.1, C72.3, C72.4, C72.5, C72.9, C73, C74.0, C74.1, C74.9, C75.0, C75.1, C75.2, C75.3, C75.4, C75.5, C76.0, C76.1, C76.2, C76.3, C76.4, C76.5, C81.0, C81.1, C81.2, C81.3, C81.4, C81.7, C81.9, C82.0, C82.1, C82.3, C82.4, C82.6, C82.7, C82.9, C83.0, C83.1, C83.3, C83.5, C83.7, C83.8, C84.0, C84.1, C84.4, C84.5, C84.6, C84.7, C84.8, C85.1, C85.2, C85.9, C88.0, C88.2, C88.3, C88.4, C90.0, C90.1, C90.2, C90.3, C91.0, C91.1, C91.3, C91.4, C91.5, C91.6, C91.7, C91.8, C91.9, C92.0, C92.1, C92.2, C92.3, C92.4, C92.5, C92.7, C92.8, C92.9. C93.0, C93.1, C93.3, C93.9, C94.0, C94.2, C94.3, C94.4, C94.5, C94.6, C94.7, C95.0, C95.1, C95.9, C96.0, C96.2, C96.4, C96.5, C96.6, C96.8) recorded within one year prior to the index date. |
| **Comedications** | |
| ACEi | Any prescription of ACEis, including captopril, enalapril, lisinopril, trandolapril, alacepril, benazepril, cilazapril, derapril, imidapril, perindopril, quinapril, and temocapril (ATC codes: C09AA01, C09AA02, C09AA03, C09AA04, C09AA06, C09AA07, C09AA08, C09AA10, C09AA12, C09AA14, C09AA16, C09AA) recorded during one year prior to the index date. |
| ARB | Any prescription of ARBs, including candesartan, losartan, valsartan, azilsartan, irbesartan, olmesartan, and telmisartan (ATC codes: C09CA01, C09CA03, C09CA04, C09CA06, C09CA07, C09CA08, C09CA09) recorded during one year prior to the index date. |
| ARNI | Any prescription of ARNI (sacubitril valsartan; ATC code: C09DX04) recorded during one year prior to the index date. |
| BB | Any prescription of beta-blockers, including bisoprolol and carvedilol (ATC codes: C07AB02, C07AG07), recorded during one year prior to the index date. |
| MRA | Any prescription of MRAs, including spironolactone and eplerenone (ATC codes: C03DA01, C03DA04), recorded during one year prior to the index date. |
| SGLT2i | Any prescription of SGLT2is, including dapagliflozin and empagliflozin (ATC codes: A10BK01, A10BK03), recorded during one year prior to the index date. |
| Oral diuretic | Any prescription of oral diuretics, including furosemide, torsemide, azosemide, hydrochlorothiazide, trichlormethiazide, benzyl hydrochlorothiazide, benzyl hydrochlorothiazide/reserpine combination, indapamide, potassium canrenoate, triamterene, and tolvaptan (ATC codes: |

| | |
|---|---|
| | C03AA03, C03AA06, C03AA, C03BA11, C03CA01, C03CA04, C03CA, C03DB02, C03XA01), recorded during one year prior to the index date. |
| Statin | Any prescription of statins, including atorvastatin, rosuvastatin, simvastatin, pravastatin, fluvastatin, and pitavastatin (ATC codes: C10AA01, C10AA03, C10AA04, C10AA05, C10AA07, C10AA08), recorded during one year prior to the index date. |
| Lipid lowering drug other than statins | Any prescription of lipid lowering drugs other than statins, including ezetimibe, fibrates, fenofibrate, gemfibrozil, alirocumab, evolocumab, bile acid sequestrants, colesevelam, and niacin (ATC codes: C10AB05, C10AX09, C10AX13, C10AX14), recorded during one year prior to the index date. |
| Antiplatelet | Any prescription of antiplatelets, including aspirin, clopidogrel, prasugrel, ticagrelor, dipyridamole, cilostazol, abciximab, and eptifibatide (ATC codes: B01AC04, B01AC07, B01AC22, B01AC23, B01AC24), recorded during one year prior to the index date. |
| Oral anticoagulant | Any prescription of oral anticoagulants, including warfarin, dabigatran, rivaroxaban, apixaban, and edoxaban (ATC codes: B01AA03, B01AE07, B01AF01, B01AF02, B01AF03), recorded during one year prior to the index date. |
| Calcium-channel blocker | Any prescription of calcium-channel blockers, including amlodipine, nifedipine, felodipine, lercanidipine, diltiazem, and verapamil (ATC codes: C08CA01, C08CA02, C08CA05, C08DA01, C08DB01), recorded during one year prior to the index date. |
| Insulin | Any prescription of insulin (ATC codes: A10AB01, A10AB04, A10AB05, A10AB06, A10AB, A10AC01, A10AC04, A10AD01, A10AD04, A10AD05, A10AD06, A10AC, A10AE04, A10AE05, A10AE06, A10AE54, A10AE56, A10AE) recorded during one year prior to the index date. |
| Biguanide | Any prescription of biguanides (metformin; ATC code: A10BA02) recorded during one year prior to the index date. |
| DPP4i | Any prescription of DPP4is, including sitagliptin, saxagliptin, vildagliptin, linagliptin, alogliptin, teneligliptin, anagliptin, trelagliptin, and omarigliptin (ATC codes: A10BH01, A10BH02, A10BH03, A10BH04, A10BH05, A10BH08, A10BH), recorded during one year prior to the index date. |
| GLP-1RA | Any prescription of GLP-1RA, including exenatide, lixisenatide, liraglutide, semaglutide, dulaglutide, and tirzepatide (ATC codes: A10BJ01, A10BJ03, A10BJ02, A10BJ05, A10BJ06, A10BX16), recorded during one year prior to the index date. |
| **Screening and diagnostic measures** | |
| Echocardiography | Assessed based on receipt codes for echocardiography (160072110, 160072210, 160072370, 160072510, 160072610, 160147110, 160147210, 160150050, 160160410, 160161710, 160165010, 160182970, 160198810, 160213010, 160218310) recorded within 90 days prior to the index date. |
| Electrocardiogram | Assessed based on receipt codes for electrocardiogram (160068410, 160068510, 160068610, 160068750, 160068850, 160150650, 160198510) recorded within 90 days prior to the index date. |
| NT-proBNP or BNP blood test | Assessed based on receipt codes for NT-proBNP or BNP blood test (160162350, 160181250) recorded within 90 days prior to the index date. |

**Table S2. Definitions of clinical events collected in this study.**

**Abbreviations:** HF, heart failure; HHF, hospitalization for heart failure.

| **Clinical s** | **Definitions** |
|---|---|
| In-hospital all-cause mortality | Occurrence of death, regardless of cause. |
| In-hospital cardiovascular death | Having at least one of the following criteria:<br>1. Patients with a diagnostic code of sudden cardiac death<br>2. Patients with a diagnostic code of unknown cause of death<br>3. Patients with the following two conditions in the discharge summary of the hospitalization:<br>- Discharge outcome is death.<br>- Myocardial infarction, HF, stroke, pulmonary embolism, peripheral artery disease, or aortic dissection is recorded as the primary diagnosis, the diagnosis leading to hospitalization, or the diagnosis which required the most medical resources. |
| HHF | Satisfying the following criteria:<br>1. Hospital admission ≥1 day and received a diagnosis of HF in the primary diagnosis, the diagnosis leading to hospitalization, or the diagnosis which required the most medical resources in the discharge summary of the hospitalization; and,<br>2. Received inpatient treatment with any of the following acute therapies:<br>- Intravenous diuretics<br>- Intravenous vasodilators<br>- Intravenous inotropes<br>- Mechanical fluid removal<br>- Intra-aortic balloon pump insertion, or<br>- Receiving oral diuretics in an inpatient setting for the first time (e.g., no history of previous oral diuretic use in the patient's records) |
| Urgent HF visit | Emergency department admission or outpatient visits with a corresponding primary diagnosis of HF and receiving intravenous diuretics, intravenous inotropes, or intravenous vasodilators. |
| Cardiovascular event | A composite event comprising non-fatal myocardial infarction, non-fatal stroke, and HHF, measured by:<br>1. The occurrence of HF, an inpatient diagnosis of myocardial infarction in the primary or discharge position, or an inpatient diagnosis of stroke in the primary or discharge position; and,<br>2. No occurrence of death within 45 days after the event. |

**Table S3. List of clinical variables included in the multivariable models.**

**Abbreviations:** ICD-10, International Classification of Diseases – 10th edition.

| Clinical variables | Definitions |
|---|---|
| **Demographic characteristics** | |
| Age | Categorical values of age: 18–44, 45–64, 64–75, or ≥75 years; an age of 18–44 years was used as the reference category. |
| Sex | Male or female; female was used as the reference category. |
| Index year | Index year 2020, 2021, 2022, or 2023; the index year 2020 was used as the reference category. |
| **Clinical characteristics** | |
| Body mass index | Body mass index <25, 25 to 30, or ≥30 kg/m$^2$; a body mass index ≥30 kg/m$^2$ was used as the reference. |
| Smoking status | Smoking status (yes or no). Smoking status was assessed based on the information recorded in the diagnostic procedure combination reporting form in the dataset. |
| Alcohol abuse | Alcohol abuse (yes or no). Alcohol abuse was defined based on diagnostic codes (ICD-10: F10.0, F10.5, F10.1, F10.2, F10.3, F10.4, F10.5, F10.6, F10.7). |
| Left-ventricular ejection fraction range | Left-ventricular ejection fraction <40, 40 to <50, or ≥50%; a left ventricular ejection fraction <40% was used as the reference. |
| **Comorbidities** | |
| Obesity | Obesity (yes or no). Obesity was defined based on diagnostic codes (ICD-10: E66.1, E66.2, E66.8, E66.9) or body mass index ≥25 kg/m$^2$ recorded within one year prior to the index date. |
| Diabetes mellitus | Diabetes mellitus (yes or no). Diabetes mellitus was defined based on diagnostic codes (ICD-10: E10, E10.0, E10.1, E10.2, E10.3, E10.4, E10.5, E10.6, E10.7, E10.9, E11, E11.0, E11.1, E11.2, E11.3, E11.4, E11.5, E11.6, E11.7, E11.9, E12, E13, E13.0, E13.1, E13.2, E13.3, E13.4, E13.5, E13.6, E13.7, E13.9, E14, E14.0, E14.1, E14.2, E14.3, E14.4, E14.5, E14.6, E14.9) or at least one prescription of diabetes-related medications during the one year prior to the index date. |
| Chronic kidney disease | Chronic kidney disease (yes or no). Chronic kidney disease was defined based on diagnostic codes (ICD-10: N18.1, N18.2, N18.3, N18.4, N18.5) or records of estimated glomerular filtration rate within one year prior to the index date. |
| Coronary artery disease | Coronary artery disease (yes or no). Coronary artery disease was defined based on diagnostic codes (ICD-10: I20.0, I20.1, I20.8, I20.9, I21.0, I21.1, I21.2, I21.3, I21.4, I21.9, I22.0, I22.1, I22.8, I22.9, I23.0, I23.1, I23.2, I23.3, I23.4, I23.5, I23.6, I23.8, I24.0, I24.1, I24.8, I24.9, I25.1, I25.2, I25.3, I25.4, I25.5, I25.6, I25.8, I25.9) within one year prior to the index date. |
| Peripheral artery disease | Peripheral artery disease (yes or no). Peripheral artery disease was defined based on diagnostic codes (ICD-10: I65.2, I70.0, I70.00, I70.10, I70.20, I70.21, I70.8, I70.80, I70.90, I70.91, I73.0, I73.1, I73.8, I73.9, I74.1, I74.2, I74.3, I74.5, I74.8, I74.9, I74.0, I74.4, I77.1, E10.5, E11.5, E13.5, E14.5) within one year prior to the index date. |
| Atrial fibrillation | Atrial fibrillation (yes or no). Atrial fibrillation was defined based on diagnostic codes (ICD-10: I48.0, I48.1, I48.2, I48.9) within one year prior to the index date. |
| Hypertension | Hypertension (yes or no). Hypertension was defined based on diagnostic codes (ICD-10: I10, I11.0, I11.9, I12.0, I12.9, I13.9, I15.0, I15.1, I15.2, I15.8, I15.9, I67.4) within one year prior to the index date. |
| Pulmonary hypertension | Pulmonary hypertension (yes or no). Pulmonary hypertension was defined based on diagnostic codes (ICD-10: I27.0, I27.2, I27.8, I27.9) |

| | |
|---|---|
| | within one year prior to the index date. |
| Resistant hypertension | Resistant hypertension (yes or no). Diagnostic codes of resistant hypertension (ICD-10: I10, I11.0, I11.9, I12.0, I12.9, I13.9, I15.0, I15.1, I15.2, I15.8, I15.9, I67.4) or concurrent use of at least four antihypertensive agents prior to the index date. |
| Cerebrovascular disease | Cerebrovascular disease (yes or no). Cerebrovascular disease was defined based on diagnostic codes (ICD-10: I60.0, I60.1, I60.2, I60.3, I60.4, I60.5, I60.6, I60.7, I60.8, I60.9, I61.0, I61.1, I61.3, I61.4, I61.5, I61.6, I61.8, I61.9, I62.0, I62.1, I62.9, I63.0, I63.1, I63.2, I63.3, I63.4, I676, I63.5, I63.6, I63.8, I63.9, I64, I65.0, I65.1, I65.2, I65.3, I66.0, I66.1, I66.2, I66.3, I66.8, I66.9, I67.0, I67.1, I67.2, I67.3, I67.4, I67.5, I67.7, I67.8, I67.9, I69.0, I69.1, I69.3, I69.4) within one year prior to the index date. |
| Chronic obstructive pulmonary disease | Chronic obstructive pulmonary disease (yes or no). Chronic obstructive pulmonary disease was defined based on diagnostic codes (ICD-10: J44.0, I44.1, I44.8, J44.9) within one year prior to the index date. |
| Dyslipidemia | Dyslipidemia (yes or no). Dyslipidemia was defined based on diagnostic codes (ICD-10: E78.0, E78.1, E78.2, E78.3, E78.4, E78.5, E78.6, E78.8, E78.9) within one year prior to the index date. |
| Renal diseases | Renal diseases (yes or no). Renal diseases were defined based on diagnostic codes (ICD-10: I12.0, N03.2, N03.3, N03.4, N03.6, N03.7, N05.2, N05.3, N05.4, N05.5, N05.6, N05.7, N18.1, N18.2, N18.3, N18.4, N18.5, N18.9, N19, N25.0, Z94.0) within one year prior to the index date. |
| Anemia | Anemia (yes or no). Anemia was defined based on diagnostic codes (ICD-10: D50.0, D50.1, D50.8, D50.9, D51.0, D51.1, D51.2, D51.3, D51.8, D51.9, D52.0, D52.1, D52.8, D52.9, D53.0, D53.1, D53.2, D53.8, D53.9, D55.0, D55.1, D55.2, D55.9, D56.0, D56.1, D56.2, D56.3, D56.4, D56.9, D57.0, D57.1, D57.2, D57.3, D57.8, D58.0, D58.1, D58.2, D58.8, D58.9, D59.0, D59.1, D59.2, D59.3, D59.4, D59.5, D59.6, D59.9, D60.0, D60.9, D61.0, D60.0, D60.1, D61.1, D61.2, D61.3, D61.9, D62, D64.0, D64.1, D64.2, D64.3, D64.4, D64.8, D64.9) or a prescription of intravenous iron or erythropoietin stimulating agents within one year prior to the index date. |
| Valvular heart disease | Valvular heart disease (yes or no). Valvular heart disease was defined based on diagnostic codes (ICD-10: I05.0, I05.1, I05.2, I05.8, I05.9, I06.0, I06.1, I06.2, I06.9, I07.0, I07.1, I07.2, I07.8, I07.9, I08.0, I08.1, I08.2, I08.3, I08.8, I08.9, I34.0, I34.1, I34.2, I34.8, I35.0, I35.1, I35.2, I35.8, I35.9, I36.0, I36.1, I36.2, I36.9, I37.0, I37.1, I37.2, I37.9, I38, Q22.0, Q22.1, Q22.2, Q22.3, Q22.4, Q22.5, Q22.6, Q22.8, Q23.0, Q23.1, Q23.2, Q23.3, Q23.4, Q23.8) within one year prior to the index date. |
| Myocardial infarction | Myocardial infarction (yes or no). Myocardial infarction was defined based on diagnostic codes (ICD-10: I21.0, I21.1, I21.2, I21.3, I21.4, I21.9, I22.0, I22.1, I22.8, I22.9, I23.0, I23.1, I23.2, I23.3, I23.4, I23.5, I23.6, I23.8, I25.2) within one year prior to the index date. |
| Depression | Depression (yes or no). Depression was defined based on diagnostic codes (ICD-10: F32.0, F32.1, F32.2, F32.3, F32.8, F32.9, F33.0, F33.1, F33.2, F33.3, F33.4, F33.9) within one year prior to the index date. |
| Dementia | Dementia (yes or no). Dementia was defined based on diagnostic codes (ICD-10: F01.0, F01.1, F01.2, F01.9, F03, F05.1, G10, G20, G30.0, G30.1, G30.8, G30.9, G31.0, G31.1, G31.8) within one year prior to the index date. |
| Stroke | Stroke (yes or no). Stroke was defined based on diagnostic codes (ICD-10: I60.0, I60.1, I60.2, I60.3, I60.4, I60.5, I60.6, I60.7, I60.8, |

| | |
|---|---|
| | I60.9, I61.0, I61.1, I61.3, I61.6, I61.4, I61.5, I61.8, I61.9, I62.0, I62.1, I62.9, I63.0, I63.1, I63.2, I63.3, I63.4, I63.5, I63.6, I63.8, I63.9) within one year prior to the index date. |
| Erectile dysfunction | Erectile dysfunction (yes or no). Erectile dysfunction was defined based on diagnostic code (ICD-10: F52.2) within one year prior to the index date. |
| Unstable angina | Unstable angina (yes or no). Unstable angina was defined based on diagnostic code (ICD-10: I20.0) within one year prior to the index date. |
| Angina pectoris | Angina pectoris (yes or no). Angina pectoris was defined based on diagnostic codes (ICD-10: I20.0, I20.1, I20.8, I20.9) within one year prior to the index date. |
| Hyperkalemia | Hyperkalemia (yes or no). Hyperkalemia was defined based on diagnostic code (ICD-10: E87.5), prescription of drugs to treat hyperkalemia (intravenous calcium chloride or calcium gluconate, hypertonic saline, insulin and glucose, beta agonists, intravenous sodium bicarbonate, or potassium binders), or laboratory data (serum potassium >5.5 mmol/L) within one year prior to the index date. |
| Hypokalemia | Hypokalemia (yes or no). Hypokalemia was defined based on diagnostic code (ICD-10: E87.6), prescription of drugs to treat hypokalemia (intravenous potassium supplements, or laboratory data (serum potassium <3.5 mmol/L) within one year prior to the index date. |
| Cancer | Cancer (yes or no). Cancer was defined based on diagnostic codes (ICD-10: C00.0, C00.1, C00.2, C00.3, C00.4, C00.6, C00.8, C00.9, C01, C02.0, C02.1, C02.2, C02.9, C03.0, C03.1, C03.9, C04.0, C04.1, C04.9, C05.0, C05.1, C05.2, C05.9, C06.0, C06.1, C06.2, C06.9, C07, C08.0, C08.1, C08.9, C09.0, C09.1, C09.9, C10.0, C10.1, C10.2, C10.3, C10.4, C10.9, C11.0, C11.1, C11.2, C11.3, C11.9, C12, C13.0, C13.1, C13.2, C13.9, C14.0, C15.0, C15.1, C15.2, C15.3, C15.4, C15.5, C15.8, C15.9, C16.0, C16.1, C16.2, C16.3, C16.4, C16.5, C16.6, C16.9, C17.0, C17.1, C17.2, C17.9, C18.0, C18.1, C18.2, C18.3, C18.4, C18.5, C18.6, C18.7, C18.9, C19, C20, C21.0, C21.1, C22.0, C22.1, C22.2, C22.3, C22.4, C22.7, C22.9, C23, C24.0, C24.1, C24.8, C24.9, C25.0, C25.1, C25.2, C25.3, C25.4, C25.7, C25.8, C25.9, C26.1, C26.9, C30.0, C30.1, C31.0, C31.1, C31.2, C31.3, C31.9, C32.0, C32.1, C32.2, C32.3, C32.9, C33, C34.0, C34.1, C34.2, C34.3, C34.8, C34.9, C37, C38.0, C38.1, C38.2, C38.3, C38.4, C40.0, C40.1, C40.2, C40.3, C41.0, C41.1, C41.2, C41.3, C41.4, C41.9, C43.0, C43.1, C43.2, C43.3, C43.4, C43.5, C43.6, C43.7, C43.8, C43.9, C45.0, C45.1, C45.2, C45.9, C46.9, C47.0, C47.1, C47.2, C47.3, C47.4, C47.5, C47.6, C47.9, C48.0, C48.1, C48.2, C49.0, C49.1, C49.2, C49.3, C49.4, C49.5, C49.6, C49.9, C50.0, C50.1, C50.1, C50.2, C50.3, C50.4, C50.5, C50.6, C50.8, C50.9, C51.0, C51.1, C51.2, C51.9, C52, C53.0, C53.1, C53.8, C53.9, C54.0, C54.1, C54.2, C54.3, C54.9, C55, C56, C57.0, C57.9, C58, C60.0, C60.1, C60.2, C60.9, C61, C62.0, C62.1, C62.9, C63.0, C63.1, C63.2, C63.7, C63.9, C64, C65, C66, C67.0, C67.1, C67.2, C67.3, C67.4, C67.5, C67.6, C67.7, C67.9, C68.0, C68.1, C69.0, C69.1, C69.2, C69.3, C69.4, C69.5, C69.6, C70.0, C70.1, C71.0, C71.1, C71.2, C71.3, C71.4, C71.5, C71.6, C71.7, C71.8, C71.9, C72.0, C72.1, C72.3, C72.4, C72.5, C72.9, C73, C74.0, C74.1, C74.9, C75.0, C75.1, C75.2, C75.3, C75.4, C75.5, C76.0, C76.1, C76.2, C76.3, C76.4, C76.5, C81.0, C81.1, C81.2, C81.3, C81.4, C81.7, C81.9, C82.0, C82.1, C82.3, C82.4, C82.6, C82.7, C82.9, C83.0, C83.1, C83.3, C83.5, C83.7, C83.8, C84.0, C84.1, C84.4, C84.5, C84.6, C84.7, C84.8, C85.1, C85.2, C85.9, C88.0, C88.2, C88.3, C88.4, C90.0, C90.1, |

| | |
|---|---|
| | C90.2, C90.3, C91.0, C91.1, C91.3, C91.4, C91.5, C91.6, C91.7, C91.8, C91.9, C92.0, C92.1, C92.2, C92.3, C92.4, C92.5, C92.7, C92.8, C92.9. C93.0, C93.1, C93.3, C93.9, C94.0, C94.2, C94.3, C94.4, C94.5, C94.6, C94.7, C95.0, C95.1, C95.9, C96.0, C96.2, C96.4, C96.5, C96.6, C96.8) within one year prior to the index date. |

**Table S4. STROBE (Strengthening the Reporting of Observational Studies in Epidemiology) Checklist of this study.**

| | Item No. | Recommendation | Page No. |
|---|---|---|---|
| **Title and abstract** | 1 | (*a*) Indicate the study design with a commonly used term in the title or abstract | 0 |
| | | (*b*) Provide in the abstract an informative and balanced summary of what was done and what was found | 1–2 |
| **Introduction** | | | |
| Background/ rationale | 2 | Explain the scientific background and rationale for the investigation being reported | 3 |
| Objectives | 3 | State the specific objectives, including any prespecified hypotheses | 4 |
| **Methods** | | | |
| Study design | 4 | Present key elements of the study design early in the paper | 4 |
| Setting | 5 | Describe the setting, locations, and relevant dates, including periods of recruitment, exposure, follow-up, and data collection | 4–5 |
| Participants | 6 | (*a*) *Cohort study*—Specify the eligibility criteria and the sources and methods of selection of participants. Describe the methods of follow-up | 5 |
| | | (*b*) For matched studies, provide the matching criteria and the number of exposed and unexposed individuals | N/A |
| Variables | 7 | Clearly define all outcomes, exposures, predictors, potential confounders, and effect modifiers. Give diagnostic criteria, if applicable | 6 |
| Data sources/ measurement | 8 | For each variable of interest, give the sources of data and details of the methods of assessment (measurement). Describe the comparability of the assessment methods if there is more than one group | 4–6 |
| Bias | 9 | Describe any efforts to address potential sources of bias | N/A |
| Study size | 10 | Explain how the study size was arrived at | 8 |
| Quantitative variables | 11 | Explain how quantitative variables were handled in the analyses. If applicable, describe which groupings were chosen and why | 6 |
| Statistical methods | 12 | (*a*) Describe all statistical methods, including those used to control for confounding | 6–7 |
| | | (*b*) Describe any methods used to examine subgroups and interactions | 6 |
| | | (*c*) Explain how missing data were addressed | 6 |
| | | (*d*) If applicable, explain how loss to follow-up was addressed | N/A |
| | | (e) Describe any sensitivity analyses | N/A |
| **Results** | | | |
| Participants | 13 | (a) Report the numbers of individuals at each stage of the study—e.g., numbers potentially eligible, examined for eligibility, confirmed eligible, included in the study, completing follow-up, and analyzed | 8–9 |
| | | (b) Give the reasons for non-participation at each stage | 8 |
| | | (c) Consider the use of a flow diagram | N/A |
| Descriptive data | 14 | (a) Give the characteristics of the study participants (e.g., demographic, clinical, social) and information on exposures and potential confounders | 8 |
| | | (b) Indicate the number of participants with missing data for each variable of interest | 10, 25–26 |
| | | (c) Summarize the follow-up time (e.g., average and total amount) | 8 |
| Outcome data | 15 | Report the numbers of outcome events or summary measures over time | 11 |
| Main results | 16 | (*a*) Give unadjusted estimates and, if applicable, confounder-adjusted estimates and their precision (e.g., 95% confidence interval). Make clear which confounders were adjusted for and why they were included | 10–11, 24 |
| | | (*b*) Report category boundaries when continuous variables were categorized | 25–26 |
| | | (*c*) If relevant, consider translating estimates of relative risk into absolute risk for a meaningful time period | N/A |
| Other analyses | 17 | Report other analyses performed—e.g., analyses of subgroups and interactions and sensitivity analyses | 10, 24 |

| | | | |
|---|---|---|---|
| **Discussion** | | | |
| Key results | 18 | Summarize the key results with reference to the study objectives | 12 |
| Limitations | 19 | Discuss the limitations of the study, taking into account sources of potential bias or imprecision. Discuss both the direction and magnitude of any potential bias | 17 |
| Interpretation | 20 | Give a cautious overall interpretation of the results considering the objectives, limitations, multiplicity of the analyses, results from similar studies, and other relevant evidence | 12–17 |
| Generalizability | 21 | Discuss the generalizability (external validity) of the study results | 17 |
| **Other information** | | | |
| Funding | 22 | Give the source of funding and the role of the funders for the present study and, if applicable, for the original study on which the present article is based | 30 |

**Table S5. Combination therapy regimen within 12 months after the first heart failure diagnosis in the overall population and stratified by left-ventricular ejection fraction category.** The stackable regimens include ACEi/ARB/ARNI, BB, MRA, and SGLT2i. Diuretics were not counted for the number of combination regimens. **Abbreviations:** ACEi, angiotensin-converting enzyme inhibitor; ARB, angiotensin receptor blocker; ARNI, angiotensin receptor blocker neprilysin inhibitor; BB, beta-blocker; HFrEF, heart failure with reduced ejection fraction; HFmrEF, heart failure with mildly reduced ejection fraction; HFpEF, heart failure with preserved ejection fraction; MRA, mineralocorticoid receptor antagonist; SGLT2i, sodium-glucose cotransporter-2 inhibitor.

| N (%) | Overall (N = 16,001) | HFrEF (N = 5,473) | HFmrEF (N = 3,053) | HFpEF (N = 7,475) |
|---|---|---|---|---|
| **None of the stackable regimens** | 4,086 (25.5) | 1,102 (20.1) | 742 (24.3) | 2,242 (30.0) |
| **One stackable regimen** | 3,905 (24.4) | 897 (16.4) | 730 (23.9) | 2,278 (30.5) |
| ACEi/ARB/ARNI | 520 (3.3) | 81 (1.5) | 103 (3.4) | 336 (4.5) |
| ACEi/ARB/ARNI + Diuretics | 882 (5.5) | 163 (3.0) | 163 (5.3) | 556 (7.4) |
| BB | 492 (3.1) | 134 (2.5) | 95 (3.1) | 263 (3.5) |
| BB + Diuretics | 792 (5.0) | 240 (4.4) | 170 (5.6) | 382 (5.1) |
| MRA | 87 (0.5) | 12 (0.2) | 12 (0.4) | 63 (0.8) |
| MRA + Diuretics | 883 (5.5) | 199 (3.6) | 151 (5.0) | 533 (7.1) |
| SGLT2i | 89 (0.6) | 22 (0.4) | 15 (0.5) | 52 (0.7) |
| SGLT2i + Diuretics | 160 (1.0) | 46 (0.8) | 21 (0.7) | 93 (1.2) |
| **Two stackable regimens** | 3,652 (22.8) | 1,130 (20.6) | 761 (24.9) | 1,761 (23.6) |
| ACEi/ARB/ARNI + BB | 467 (2.9) | 122 (2.2) | 97 (3.2) | 248 (3.3) |
| ACEi/ARB/ARNI + BB + Diuretics | 915 (5.7) | 327 (6.0) | 206 (6.8) | 382 (5.1) |
| ACEi/ARB/ARNI + MRA | 80 (0.5) | 16 (0.3) | 20 (0.7) | 44 (0.6) |
| ACEi/ARB/ARNI + MRA + Diuretics | 717 (4.5) | 185 (3.4) | 150 (4.9) | 382 (5.1) |
| ACEi/ARB/ARNI + SGLT2i | 91 (0.6) | 16 (0.3) | 15 (0.5) | 60 (0.8) |
| ACEi/ARB/ARNI + SGLT2i + Diuretics | 224 (1.4) | 58 (1.1) | 38 (1.2) | 128 (1.7) |
| BB + MRA | 73 (0.5) | 31 (0.6) | 8 (0.3) | 34 (0.5) |
| BB + MRA + Diuretics | 702 (4.4) | 253 (4.6) | 148 (4.9) | 301 (4.0) |
| BB + SGLT2i | 64 (0.4) | 19 (0.4) | 17 (0.6) | 28 (0.4) |
| BB + SGLT2i + Diuretics | 187 (1.2) | 69 (1.3) | 42 (1.4) | 76 (1.0) |
| MRA + SGLT2i | 25 (0.2) | 6 (0.1) | 4 (0.1) | 15 (0.2) |
| MRA + SGLT2i + Diuretics | 107 (0.7) | 28 (0.5) | 16 (0.5) | 63 (0.8) |
| **Three stackable regimens** | 2,825 (17.7) | 1,338 (24.4) | 543 (17.8) | 944 (12.6) |
| ACEi/ARB/ARNI + BB + MRA | 187 (1.2) | 91 (1.7) | 39 (1.3) | 57 (0.8) |
| ACEi/ARB/ARNI + BB + MRA + Diuretics | 1,284 (8.0) | 633 (11.6) | 240 (7.9) | 411 (5.5) |
| ACEi/ARB/ARNI + BB + SGLT2i | 209 (1.3) | 106 (1.9) | 45 (1.5) | 58 (0.8) |
| ACEi/ARB/ARNI + BB + SGLT2i + Diuretics | 480 (3.0) | 240 (4.4) | 92 (3.0) | 148 (2.0) |
| ACEi/ARB/ARNI + MRA + SGLT2i | 55 (0.3) | 20 (0.4) | 7 (0.2) | 28 (0.4) |
| ACEi/ARB/ARNI + MRA + SGLT2i + Diuretics | 295 (1.8) | 104 (1.9) | 50 (1.6) | 141 (1.9) |
| BB + MRA + SGLT2i | 57 (0.4) | 21 (0.4) | 16 (0.5) | 20 (0.3) |
| BB + MRA + SGLT2i + Diuretics | 258 (1.6) | 123 (2.3) | 54 (1.8) | 81 (1.1) |
| **Four stackable regimens** | 1,533 (9.6) | 1,006 (18.4) | 277 (9.1) | 250 (3.3) |
| ACEi/ARB/ARNI + BB + MRA + SGLT2i | 338 (2.1) | 232 (4.2) | 52 (1.7) | 54 (0.7) |
| ACEi/ARB/ARNI + BB + MRA + SGLT2i + Diuretics | 1,195 (7.5) | 774 (14.1) | 225 (7.4) | 196 (2.6) |

**Table S6. Prescription rates of SGLT2i and ARNI in the annual cross-sectional cohorts of HFmrEF/pEF in each calendar year between 2020 and 2024.** Patients with at least one ICD-10 diagnostic code of HF, LVEF ≥40%, at least ≥18 years of age on January 1, and having at least one day of follow-up were identified and included in the analysis in each calendar year. Therefore, prevalent HF patients are included in the analysis. *Data period was until July 31, 2024. **Abbreviations:** ARNI, angiotensin receptor blocker neprilysin inhibitor; HFmrEF, heart failure with mildly reduced ejection fraction; HFpEF, heart failure with preserved ejection fraction; ICD-10, International Classification of Diseases–10$^{th}$ revision; LVEF, left-ventricular ejection fraction; SGLT2i, sodium-glucose cotransporter-2 inhibitor.

| N (%) | 2020 (N = 1) | 2021 (N = 4) | 2022 (N = 2,428) | 2023 (N = 8,686) | 2024* (N = 7,394) |
|---|---|---|---|---|---|
| SGLT2i | 0 (0) | 1 (25) | 601 (24.8) | 3,064 (35.3) | 2,893 (39.1) |
| ARNI | 0 (0) | 0 (0) | 485 (20.0) | 2,445 (28.2) | 2,441 (33.0) |

**Figure S1. Sankey diagrams of the patterns of treatment continuations of pharmacological treatments in the overall population and stratified by left-ventricular ejection fraction category.** Panel (A) shows the results for the overall population. Panels (B), (C), and (D) show the results for the HFrEF, HFmrEF, and HFpEF subgroups, respectively. Treatment continuations were grouped into the following categories: Continue - no changes occurred in use of the medication class; Add-on - other HF medication class(es) were added during use of the medication class; Discontinue - use of the medication class was discontinued; Decrease - use of the medication class was continued, but at least one other medication was discontinued; and Censor - cases where the patient's observation was terminated. **Abbreviations:** ACEi, angiotensin-converting enzyme inhibitor; ARB, angiotensin receptor blocker; ARNI, angiotensin receptor blocker neprilysin inhibitor; BB, beta-blocker; HF, heart failure; HFrEF, heart failure with reduced ejection fraction; HFmrEF, heart failure with mildly reduced ejection fraction; HFpEF, heart failure with preserved ejection fraction; MRA, mineralocorticoid receptor antagonist; SGLT2i, sodium-glucose cotransporter-2 inhibitor.

**(A)**

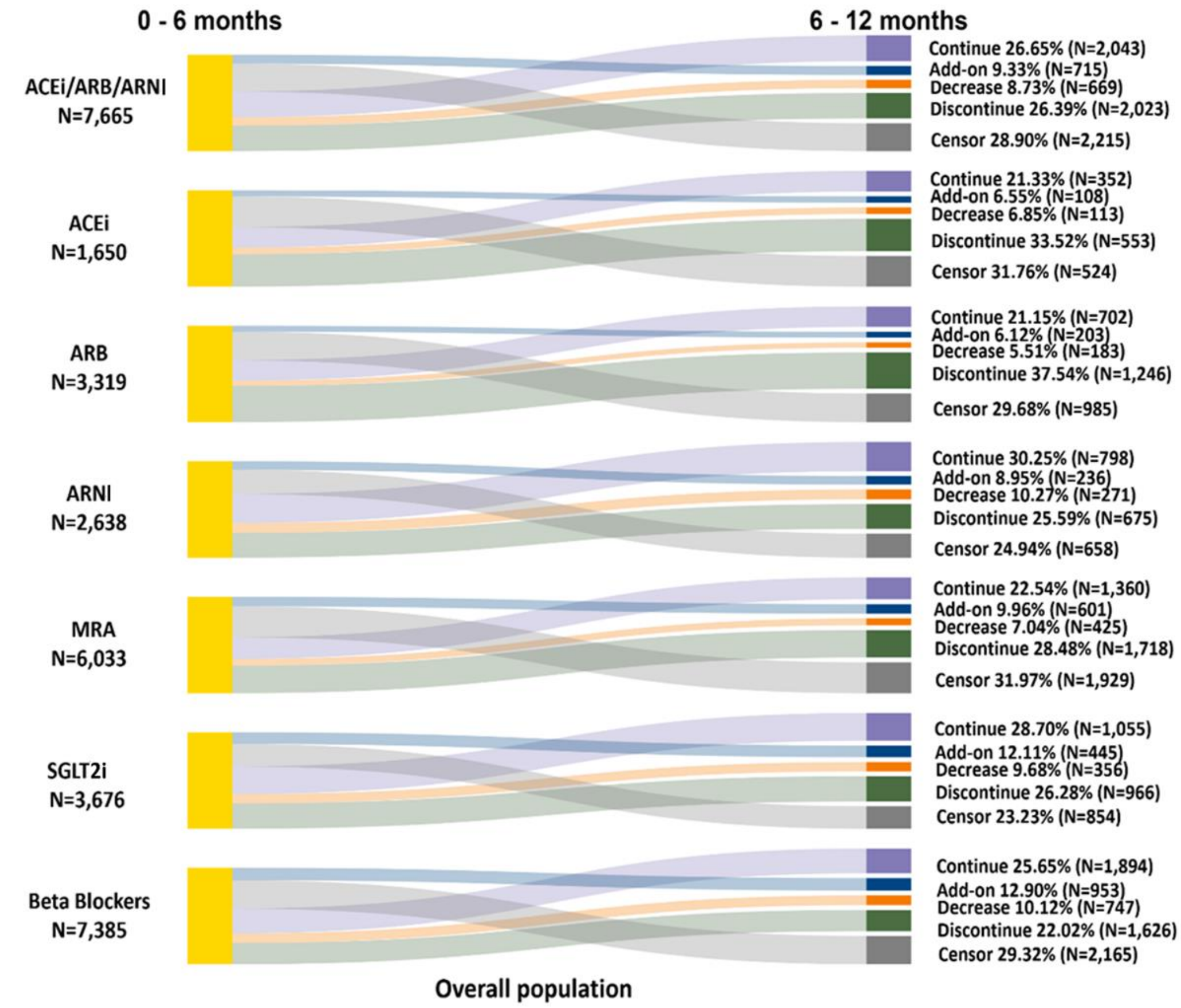

# (B)

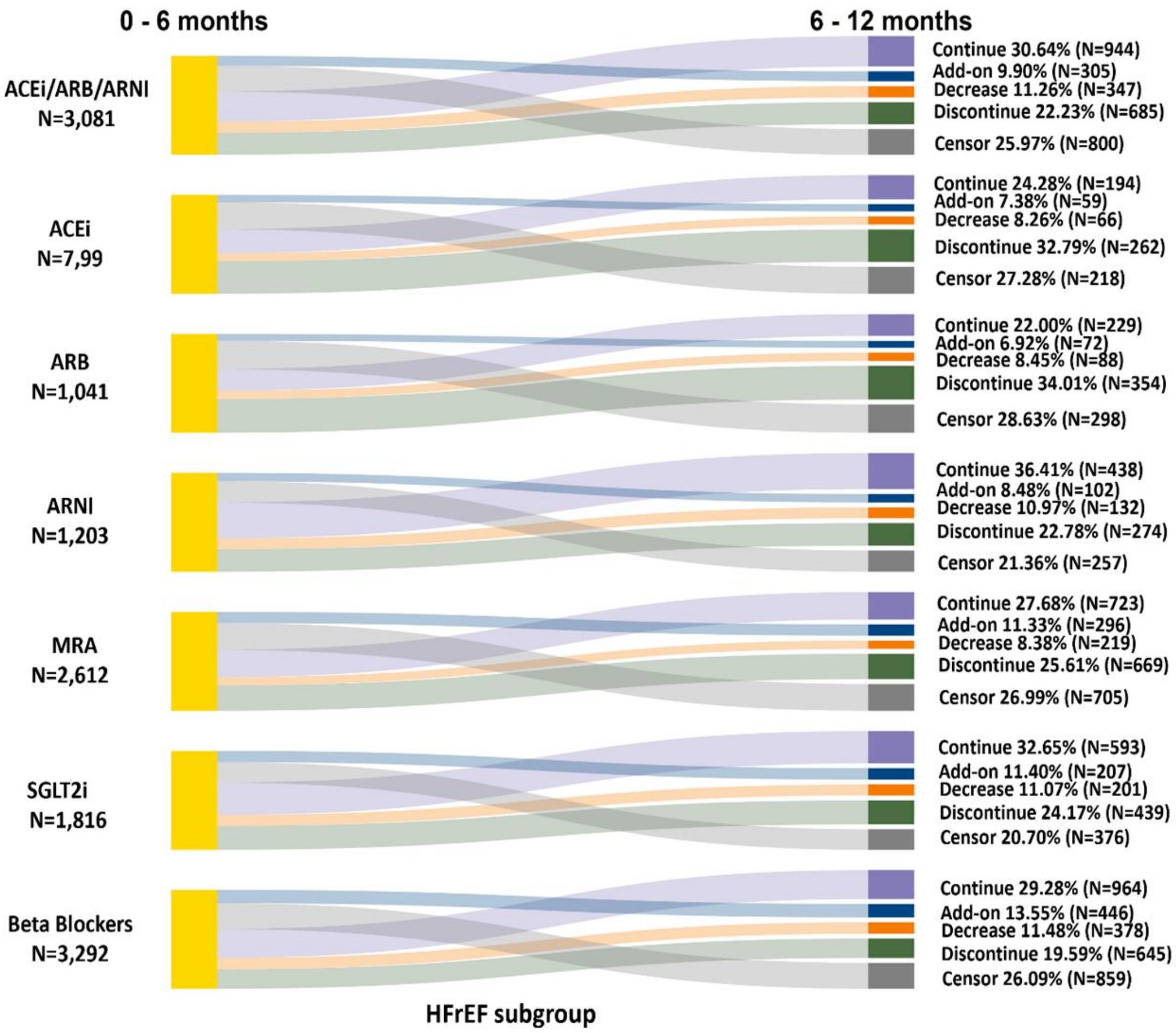
0 - 6 months
6 - 12 months
ACEi/ARB/ARNI N=3,081
Continue 30.64% (N=944)
Add-on 9.90% (N=305)
Decrease 11.26% (N=347)
Discontinue 22.23% (N=685)
Censor 25.97% (N=800)
ACEi N=7,99
Continue 24.28% (N=194)
Add-on 7.38% (N=59)
Decrease 8.26% (N=66)
Discontinue 32.79% (N=262)
Censor 27.28% (N=218)
ARB N=1,041
Continue 22.00% (N=229)
Add-on 6.92% (N=72)
Decrease 8.45% (N=88)
Discontinue 34.01% (N=354)
Censor 28.63% (N=298)
ARNI N=1,203
Continue 36.41% (N=438)
Add-on 8.48% (N=102)
Decrease 10.97% (N=132)
Discontinue 22.78% (N=274)
Censor 21.36% (N=257)
MRA N=2,612
Continue 27.68% (N=723)
Add-on 11.33% (N=296)
Decrease 8.38% (N=219)
Discontinue 25.61% (N=669)
Censor 26.99% (N=705)
SGLT2i N=1,816
Continue 32.65% (N=593)
Add-on 11.40% (N=207)
Decrease 11.07% (N=201)
Discontinue 24.17% (N=439)
Censor 20.70% (N=376)
Beta Blockers N=3,292
Continue 29.28% (N=964)
Add-on 13.55% (N=446)
Decrease 11.48% (N=378)
Discontinue 19.59% (N=645)
Censor 26.09% (N=859)
HFrEF subgroup

**(C)**

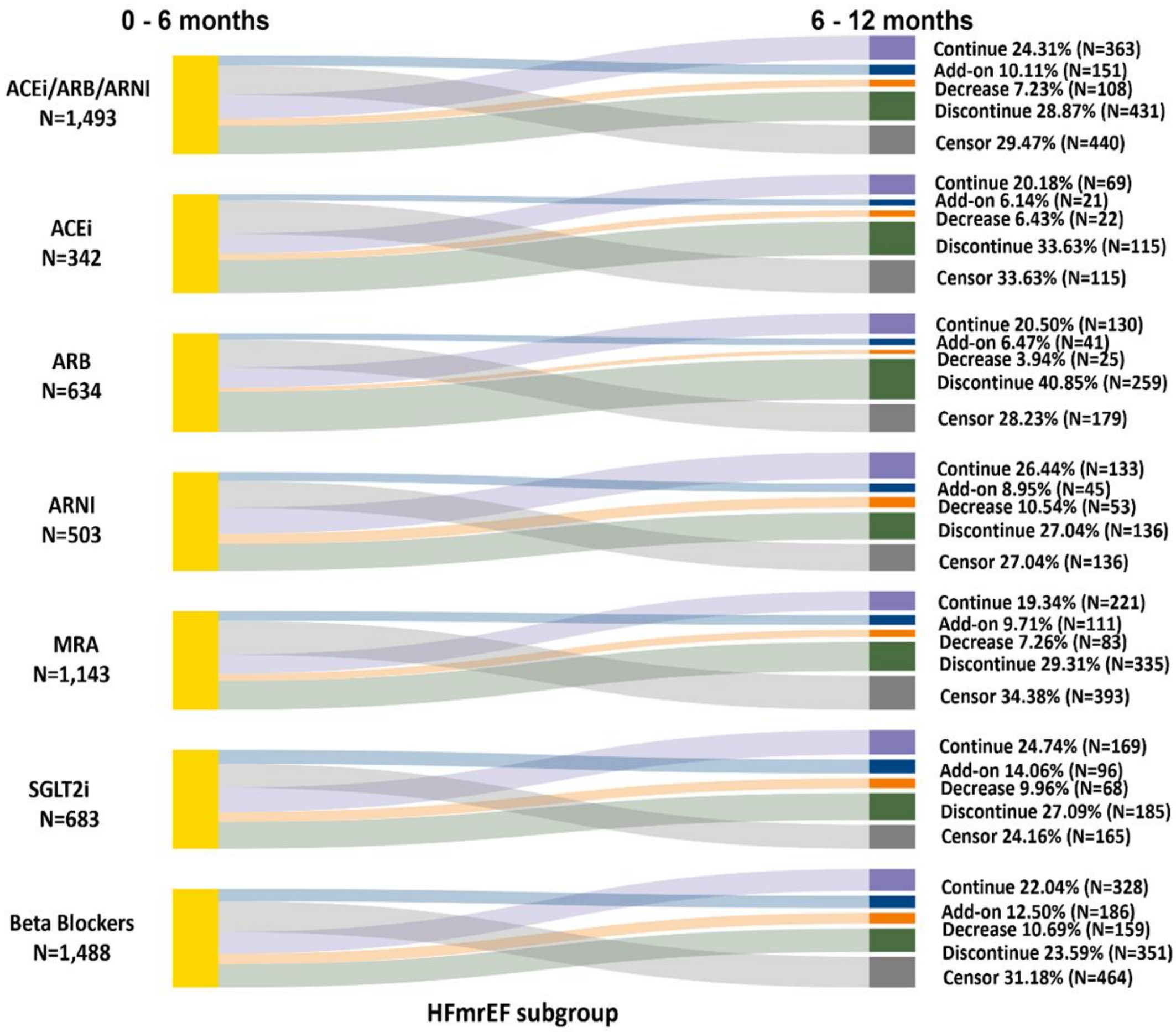
0 - 6 months
6 - 12 months
ACEi/ARB/ARNI
N=1,493
Continue 24.31% (N=363)
Add-on 10.11% (N=151)
Decrease 7.23% (N=108)
Discontinue 28.87% (N=431)
Censor 29.47% (N=440)
ACEi
N=342
Continue 20.18% (N=69)
Add-on 6.14% (N=21)
Decrease 6.43% (N=22)
Discontinue 33.63% (N=115)
Censor 33.63% (N=115)
ARB
N=634
Continue 20.50% (N=130)
Add-on 6.47% (N=41)
Decrease 3.94% (N=25)
Discontinue 40.85% (N=259)
Censor 28.23% (N=179)
ARNI
N=503
Continue 26.44% (N=133)
Add-on 8.95% (N=45)
Decrease 10.54% (N=53)
Discontinue 27.04% (N=136)
Censor 27.04% (N=136)
MRA
N=1,143
Continue 19.34% (N=221)
Add-on 9.71% (N=111)
Decrease 7.26% (N=83)
Discontinue 29.31% (N=335)
Censor 34.38% (N=393)
SGLT2i
N=683
Continue 24.74% (N=169)
Add-on 14.06% (N=96)
Decrease 9.96% (N=68)
Discontinue 27.09% (N=185)
Censor 24.16% (N=165)
Beta Blockers
N=1,488
Continue 22.04% (N=328)
Add-on 12.50% (N=186)
Decrease 10.69% (N=159)
Discontinue 23.59% (N=351)
Censor 31.18% (N=464)
HFmrEF subgroup

(D)

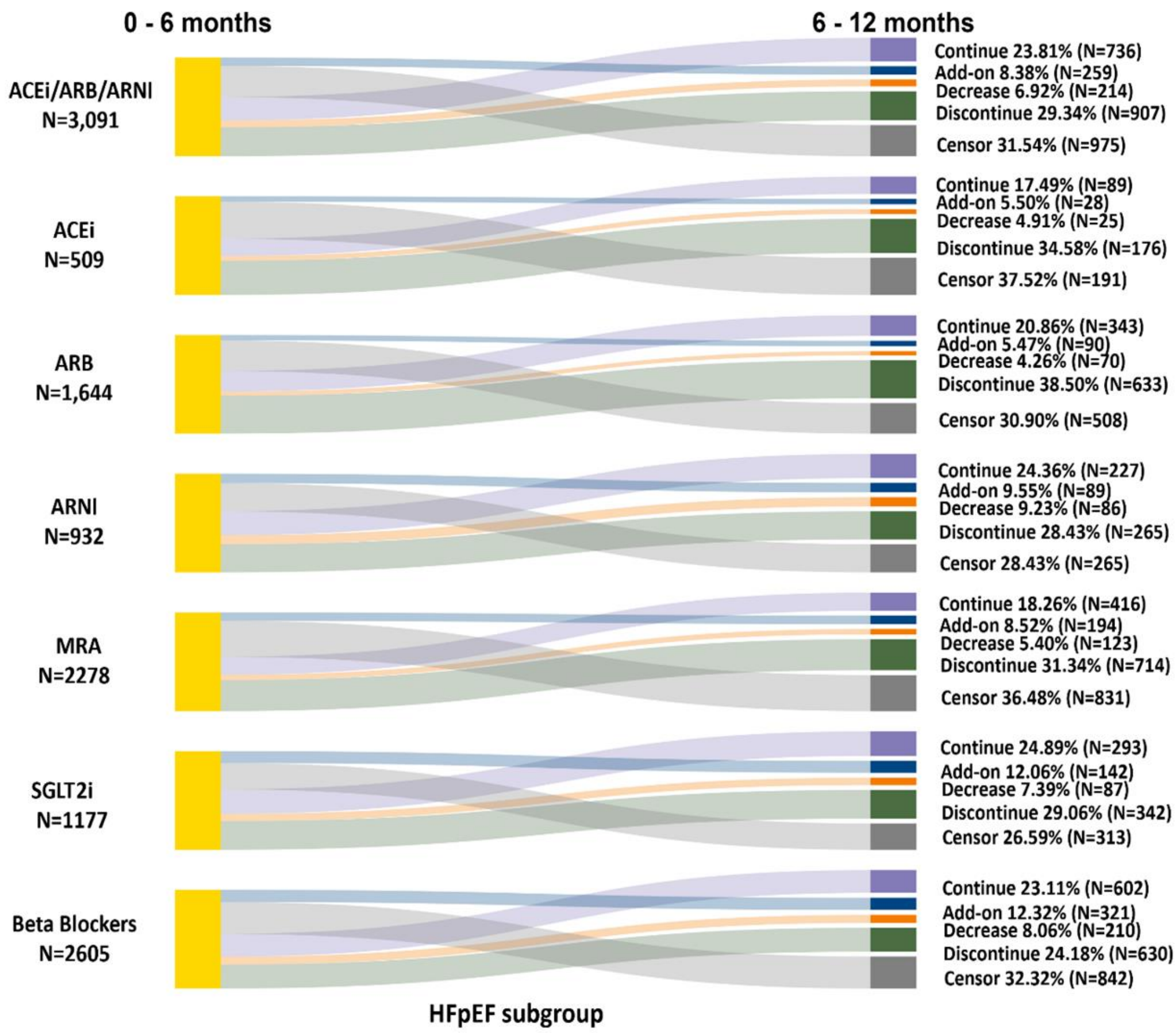
0 - 6 months
6 - 12 months
ACEi/ARB/ARNI N=3,091
Continue 23.81% (N=736)
Add-on 8.38% (N=259)
Decrease 6.92% (N=214)
Discontinue 29.34% (N=907)
Censor 31.54% (N=975)
ACEi N=509
Continue 17.49% (N=89)
Add-on 5.50% (N=28)
Decrease 4.91% (N=25)
Discontinue 34.58% (N=176)
Censor 37.52% (N=191)
ARB N=1,644
Continue 20.86% (N=343)
Add-on 5.47% (N=90)
Decrease 4.26% (N=70)
Discontinue 38.50% (N=633)
Censor 30.90% (N=508)
ARNI N=932
Continue 24.36% (N=227)
Add-on 9.55% (N=89)
Decrease 9.23% (N=86)
Discontinue 28.43% (N=265)
Censor 28.43% (N=265)
MRA N=2278
Continue 18.26% (N=416)
Add-on 8.52% (N=194)
Decrease 5.40% (N=123)
Discontinue 31.34% (N=714)
Censor 36.48% (N=831)
SGLT2i N=1177
Continue 24.89% (N=293)
Add-on 12.06% (N=142)
Decrease 7.39% (N=87)
Discontinue 29.06% (N=342)
Censor 26.59% (N=313)
Beta Blockers N=2605
Continue 23.11% (N=602)
Add-on 12.32% (N=321)
Decrease 8.06% (N=210)
Discontinue 24.18% (N=630)
Censor 32.32% (N=842)
HFpEF subgroup

**Figure S2. Cumulative incidence curves of clinical events in patients stratified by left-ventricular ejection fraction category.** Panels (A), (B), (C), (D), and (E) show the results for in-hospital all-cause mortality, in-hospital cardiovascular death, HHF, urgent HF visit, and cardiovascular event, respectively. **Abbreviations:** HF, heart failure; HHF, hospitalization for heart failure; HFrEF, heart failure with reduced ejection fraction; HFmrEF, heart failure with mildly reduced ejection fraction; HFpEF, heart failure with preserved ejection fraction.

**(A)**

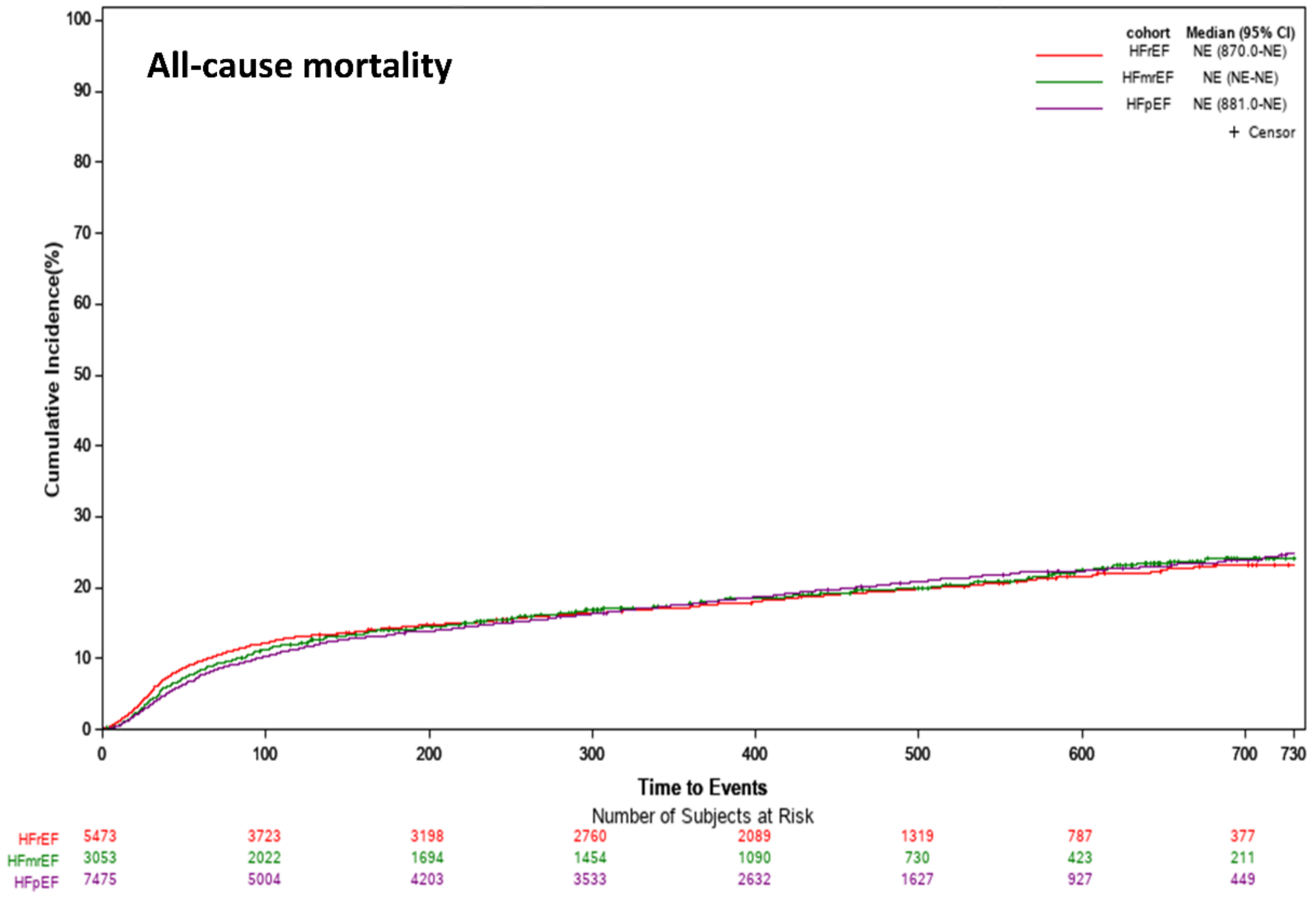


**(B)**

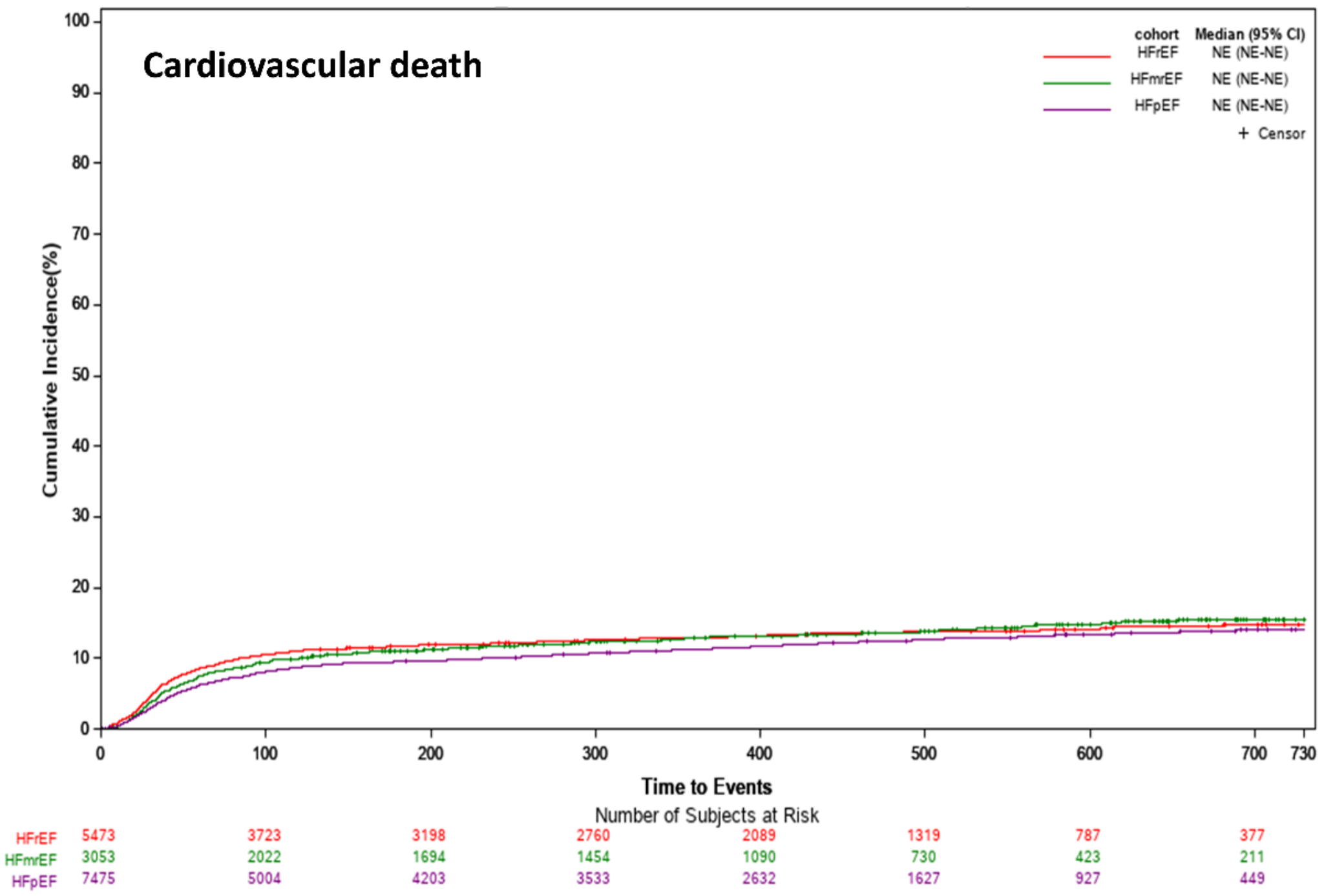

**(C)**

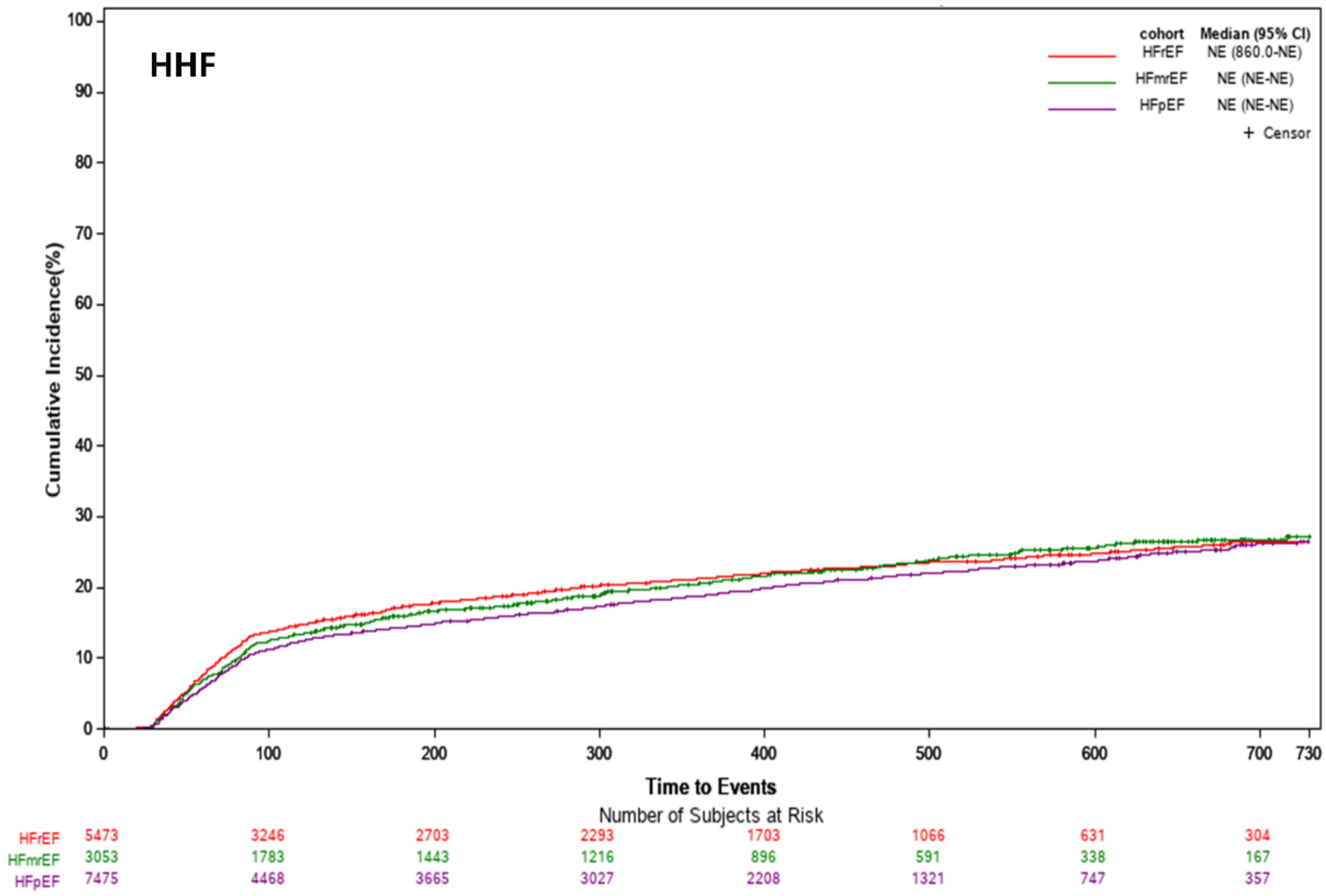
HHF
cohort Median (95% CI)
HFrEF NE (860.0-NE)
HFmrEF NE (NE-NE)
HFpEF NE (NE-NE)
+ Censor
Cumulative Incidence(%)
0 10 20 30 40 50 60 70 80 90 100
0 100 200 300 400 500 600 700 730
Time to Events
Number of Subjects at Risk
HFrEF 5473 3246 2703 2293 1703 1066 631 304
HFmrEF 3053 1783 1443 1216 896 591 338 167
HFpEF 7475 4468 3665 3027 2208 1321 747 357

**(D)**

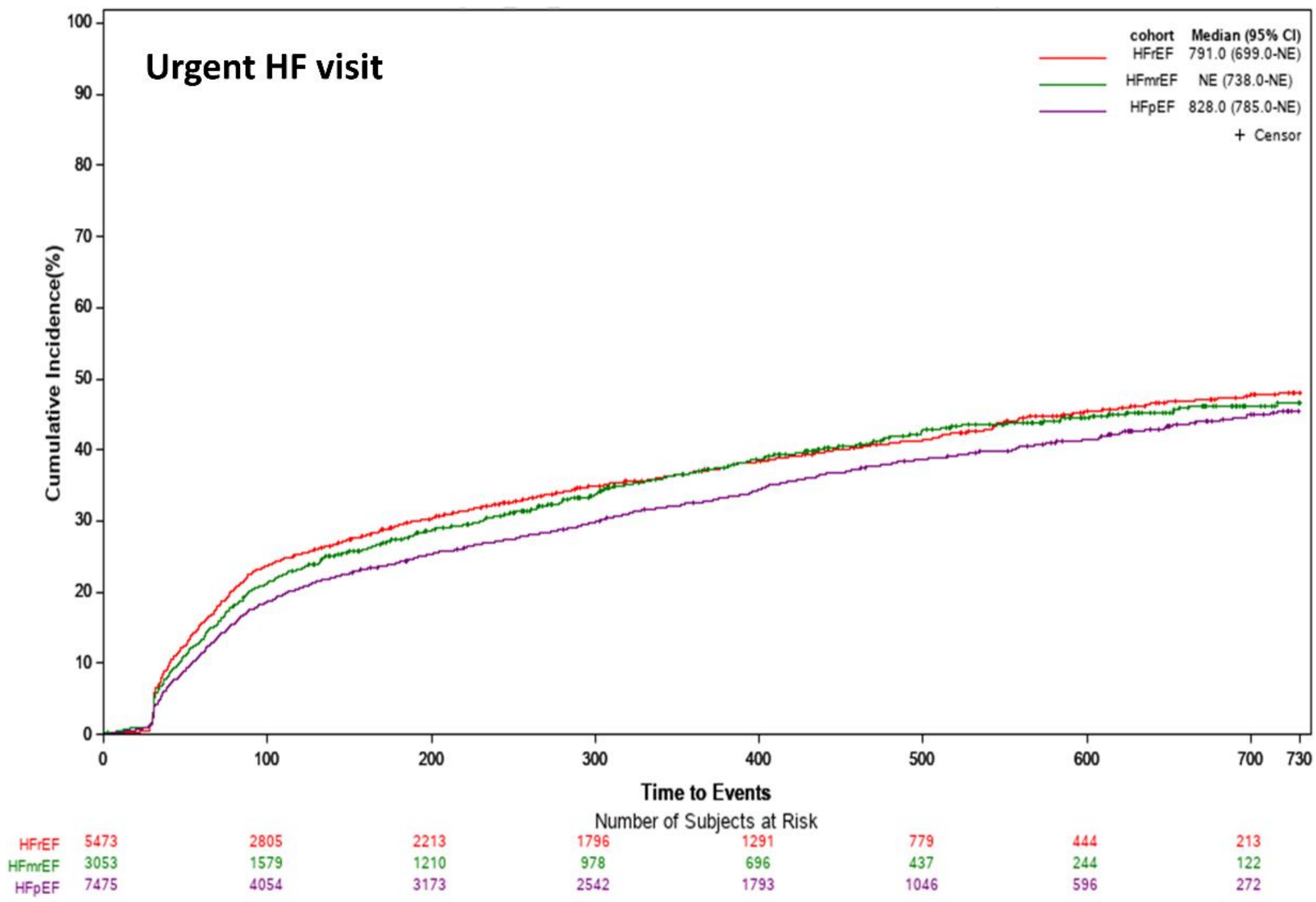
Urgent HF visit
cohort Median (95% CI)
HFrEF 791.0 (699.0-NE)
HFmrEF NE (738.0-NE)
HFpEF 828.0 (785.0-NE)
+ Censor
Cumulative Incidence(%)
0 10 20 30 40 50 60 70 80 90 100
0 100 200 300 400 500 600 700 730
Time to Events
Number of Subjects at Risk
HFrEF 5473 2805 2213 1796 1291 779 444 213
HFmrEF 3053 1579 1210 978 696 437 244 122
HFpEF 7475 4054 3173 2542 1793 1046 596 272

**(E)**

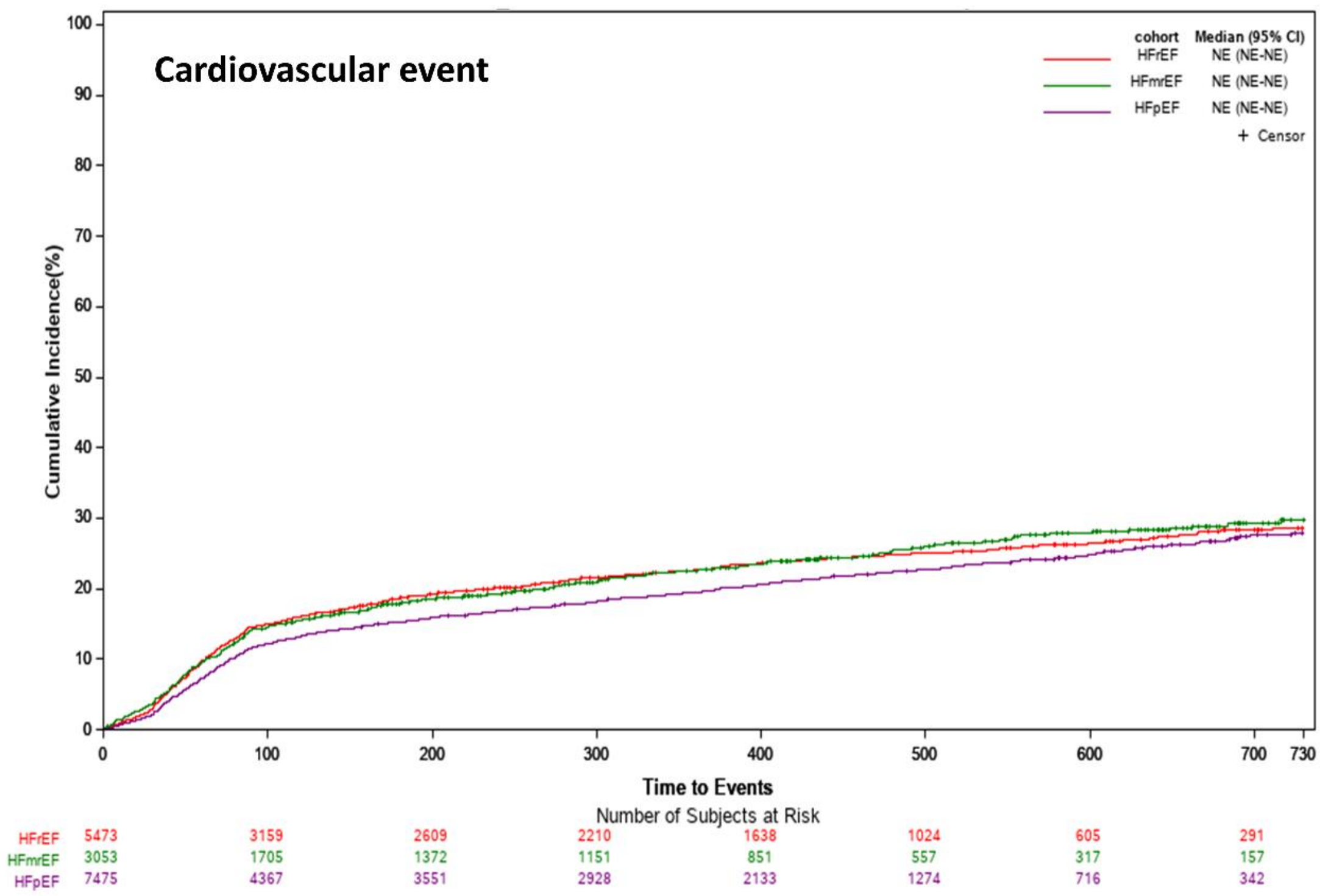

Cardiovascular event
cohort Median (95% CI)
HFrEF NE (NE-NE)
HFmrEF NE (NE-NE)
HFpEF NE (NE-NE)
+ Censor
Cumulative Incidence(%)
100
90
80
70
60
50
40
30
20
10
0
0 100 200 300 400 500 600 700 730
Time to Events
Number of Subjects at Risk
HFrEF 5473 3159 2609 2210 1638 1024 605 291
HFmrEF 3053 1705 1372 1151 851 557 317 157
HFpEF 7475 4367 3551 2928 2133 1274 716 342
